# Pre-existing antibody and T cell responses to SaCas9, AsCas12a and CasΦ are comparable in naïve individuals

**DOI:** 10.1038/s41467-026-75015-7

**Published:** 2026-07-01

**Authors:** Vijaya L. Simhadri, Pratima Bajgain, Sharon Vijayanand, Wojciech Jankowski, Atul Rawal, Lauren Thijs, Thomas Van Doninck, Kurt Boonen, Sofie Pattyn, Elise Pepermans, Zuben E. Sauna

**Affiliations:** 1 Division of Hemostasis, Office of Plasma Protein Therapeutics, Office of Therapeutic Products, Center for Biologics Evaluation and Research, Food and Drug Administration, Silver Spring, MD USA; 2 ImmuneSpec, Niel, Belgium; 3 IQVIA In vitro Immunology, Charleroi, Belgium

**Keywords:** Antigen processing and presentation, Translational immunology, CRISPR-Cas9 genome editing, Adaptive immunity

## Abstract

Cas9 proteins are derived from human pathogens and are immunogenic, thereby raising potential safety concerns and limiting clinical effectiveness when using Cas9 as a gene-editing tool. Cas orthologs developed from organisms not directly associated with human infections may thus be safer. Here we compare the immunogenicity risk of SaCas9 (derived from the human pathogen, *Staphylococcus aureus*), AsCas12a (derived from the human commensal, *Acidaminococcus* sp.) and CasΦ (derived from a bacteria phage, Biggiephage). Ex vivo and in vitro analyses show that SaCas9, AsCas12a and CasΦ are recognized similarly by antibodies and T cells from unimmunized individuals. Using mass-spectrometry to identify MHC-I-bound peptides, we find SaCas9, AsCas12a and CasΦ peptides presented on 9 MHC-I proteins commonly found in the North American population. Our results thus indicate that AsCas12a and CasΦ do not present a less immunogenic alternative to Cas9, and underscore the need for systematic immunogenicity evaluation of all Cas proteins intended for clinical use.

## Abstract (web summary)

Cas9 protein from human-infecting bacteria has been used for in vivo gene editing, but immunogenicity of Cas9 presents potential risk and roadblock for translational applications. Here the authors show that Cas9 orthologs from non-human-infecting sources such bacteriophage are equally recognized by naïve immune systems, so do not represent better alternatives vis-à-vis immunogenicity.

## Introduction

CRISPR-Cas based genome editing holds immense promise in treating diverse human diseases^1^. These range from rare and ultra-rare genetic diseases to human cancers^2^. The technology uses a nuclease (Cas) to generate breaks in DNA sequences^3^. These cuts in the DNA occur only when the Cas is complexed with a short guide RNA^3,4^. Thus, site specificity can be achieved by using appropriate guide RNA sequences^4^.

Broadly, methods that seek to clinically exploit Cas-based genome editing rely on two approaches: (i) Transplantation of cells that have been edited ex vivo^5,6^. (ii) Editing cells in vivo following vector-based delivery of the CRISPR-Cas system^7,8^. Either viral vectors or nanoparticles can be used to deliver the cargo into cells^9^. The Cas protein moreover may be introduced either as a DNA^10,11^, mRNA^12^, or protein^13^.

Cas9 proteins derived from human pathogens (originating from *Streptococcus pyogenes* [*S. pyogenes*] and *Staphylococcus aureus* [*S. aureus*] are the most extensively studied CRISPR-Cas gene editors^14–16^. These proteins have been successfully used for both ex vivo and in vivo gene editing^15,17^. An important hurdle when developing and licensing a protein for clinical applications is the potential for immune responses to the protein^18^. Humans are exposed to both *S. pyogenes* and *S. aureus* and antibodies to both microbes have been identified in a large fraction of the adult human population^19^. These observations led to the surmise that there could be preexisting immunity to Cas9 proteins. Multiple studies have now demonstrated both T and B cell immunity to Cas9 derived from either *S. pyogenes* (SpCas9)^20^ or *S. aureus* (SaCas9)^21,22^.

While Cas9 was the first Cas to be technologically exploited in gene editing, numerous alternatives have been reported since. Based on the structural and functional characteristics of the Cas proteins, the CRISPR-Cas system is classified into two major classes: Class I (Cas3 and Cas10) and II (Cas9, Cas12, and Cas13)^8,23,24^. Class I system consists of multi-subunit effector Cas protein complexes, while Class II systems use single Cas protein effectors. Due to its structural simplicity and mechanism of action, the Cas proteins of the Class II system, particularly SpCas9 and SaCas9 are well studied and extensively used to perform genetic modifications^15,16,25^. Recently, many Cas orthologs, and related effector proteins from diverse sources have been technologically exploited for gene editing^26^. One example is the CRISPR-CasΦ (Cas12j) from the huge bacteriophage. As CasΦ is obtained from a bacteriophage it has been speculated that this protein would not show preexisting immunity in humans^27^. However, the absence of potential human exposure may not be a sufficient condition for the absence of preexisting antibodies. For instance, antibodies targeting *Ruminococcus flavefaciens* Cas13d (which is not known to colonize humans), have been detected and have a prevalence that is comparable to that observed for Cas9 proteins^28^. Different Cas proteins are studied for their advantages with respect to packaging within vectors, accuracy and precision of gene-editing, frequency of off-target effects etc^26^. It is important that these molecules are also compared with respect to their immunogenicity risk.

Here, to address whether the source organism of a Cas protein predicts its immunogenicity, we employ a multi-pronged strategy that integrates B cell and T cell response profiling with a novel MHC-I–based MAPPs workflow using monoallelic cell lines transduced with each Cas gene. We find that pre-existing antibodies and memory T cell responses are prevalent across SaCas9, AsCas12a, and CasΦ. We identify MHC-I–bound T cell epitopes distributed across functionally important regions of each protein. These findings challenge the assumption that phylogenetic distance from human pathogens is a reliable surrogate for reduced immunogenicity risk. Our findings underscore the need for rigorous, empirical immunogenicity assessment for all Cas orthologs under clinical development to ensure the safe and effective translation of CRISPR-based therapies to the clinic.

## Results

### Curation of donor pool for studying B and T cell responses to diverse Cas proteins

**Fig. 1:**
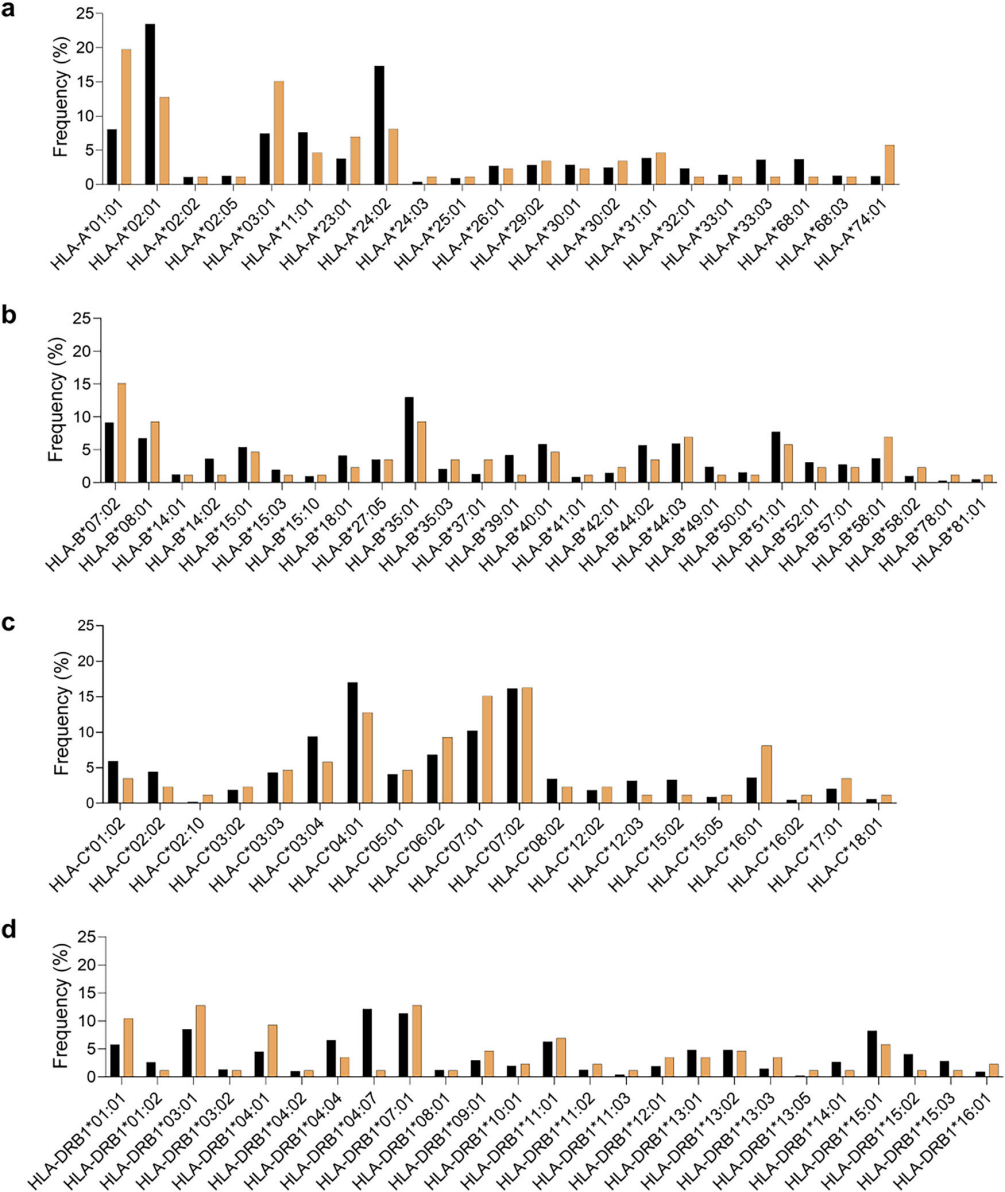
Distribution of HLA Class I and Class II variants in the donor pool used to measure B cell and T cell responses using ELISA and ELISpot assay respectively. The frequency for each allele in North American Population was obtained from The Allele Frequency Net Database. Total population coverage was obtained by adding population coverage of each allele used in the study. **a** Comparison of MHC class I, HLA-A allele frequencies in North American Population and cohort of donors used in assays. **b** Comparison of MHC Class I, HLA-B allele frequencies in North American Population and cohort of donors used in assays. **c** Comparison of MHC Class I, HLA-C allele frequencies in North American Population and cohort of donors used in assays. **d** Comparison of MHC Class II, HLA-DRB1 allele frequencies in North American Population and cohort of donors used in assays. Normalized North American population frequency (Black) and Donor cohort frequency (Orange). Source data are provided as a Source Data file.

Here, we sought to estimate the pre-existing immune responses against three Cas proteins (SaCas9, AsCas12a, and CasΦ in the North American population. The Major Histocompatibility Complex (MHC) proteins (also called Human leukocyte antigen (HLA)) play an important role in determining which individuals develop an immune response to protein-based therapies^29,30^. The binding of foreign peptides and their presentation by MHC proteins represents a necessary (albeit not sufficient) condition for eliciting an immune response. The MHC genes are the most polymorphic in the human genome^29,31^. It is therefore essential that the donor pool has a distribution of HLA variants that is comparable to that of the population. Using a previously described algorithm^32^, we selected 43 donors that show a distribution of HLA Class I (HLA-I) and Class II (HLA-II) variants that is comparable to that found in the North American population (Fig. 1). Our algorithm utilizes simulated annealing to optimizes a cohort to match the HLA distribution of the target population, a cohort of donors selected using this algorithm performed 7 standard deviations better than 10 million randomly picked cohorts. The same donor pool was used to measure B and T cell responses to all three Cas proteins.

### Detection of pre-existing antibodies to Cas proteins

**Fig. 2:**
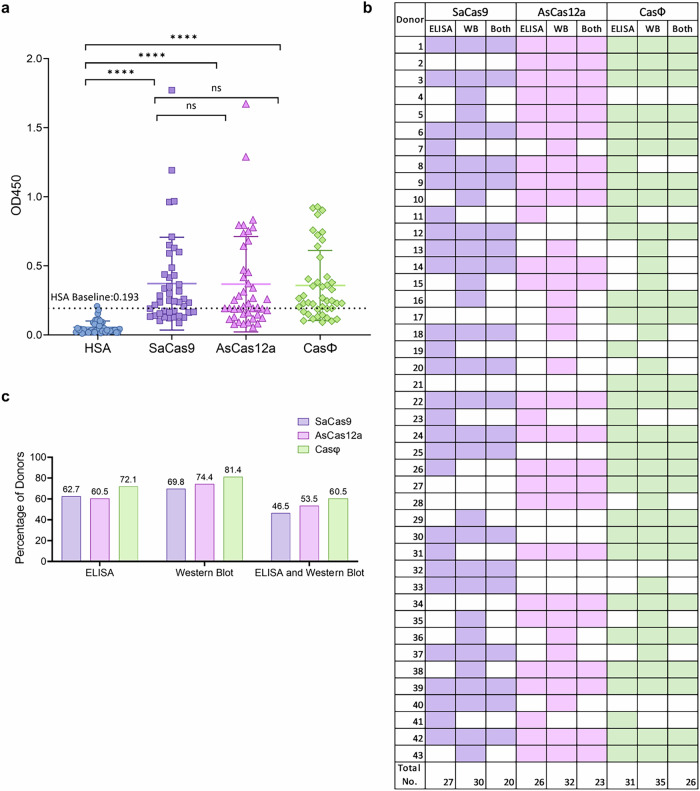
Detection of pre-existing antibodies to different Cas proteins. a Antibodies against Human Serum Albumin (HSA, negative control), SaCas9, AsCas12a, and CasΦ in 43 healthy human donors were detected using ELISA. The baseline (0.193) was determined as mean value of HSA + 3 SD. All the donors above the baseline (dotted line) were considered antibody positive. Statistical significance was analyzed using a two-tailed paired student t-test and *P*-values were determined. *P ≤ 0.05; **P ≤ 0.01; ***P ≤ 0.001; ****P ≤ 0.0001, ns – non-significant. Data is presented as mean ± SD. Each point represents one independent donor (*n* = 43 biological replicates). Technical replicate measurements were averaged prior to statistical analysis. Detailed statistics are provided in the Supplementary Table 4. **b** Individual donor response to ELISA and western blot for each Cas protein. **c** Percentage of donors that responded in ELISA, western blot, and both assays for each Cas protein are depicted above the violet, pink and green bars respectively. Source data are provided as a Source Data file.

To determine prevalence of pre-existing anti-SaCas9, anti-AsCas12a and anti-CasΦ antibodies in the healthy human population, we performed an enzyme-linked immunosorbent assay (ELISA) with human serum obtained from the 43 healthy donors described in Fig. 1. We performed the ELISA using serial dilutions of donor sera after immobilizing each of the 3 Cas proteins. The purity and the endotoxin levels of the 3 Cas proteins were evaluated and are reported in Supplementary Fig. 1. The EC50 values for each donor-Cas protein combination were obtained (Suppl. Table 1 and Suppl. Fig. 2). Previous studies have detected pre-existing antibodies against SaCas9^19,21^. Consistent with these findings, sera obtained from the donors showed significantly (*P* < 0.0001) higher levels of anti-SaCas9 antibodies compared to the HSA control (Fig. 2a). Moreover, these sera samples also showed significantly (*P* < 0.0001) higher levels of anti-AsCas12a and CasΦ antibodies compared to the HSA control (Fig. 2a). While sera from these donors showed significantly higher levels of antibodies to SaCas9, AsCas12a and CasΦ compared to the HSA control there were no significant differences between anti-SaCas9, AsCas12a and CasΦ antibodies (Fig. 2a). To estimate the prevalence of anti-Cas antibodies in the North American population we established a cut-point, based on standard methodologies (see Methods) to determine if serum from an individual donor contained antibodies to each of the Cas proteins. Based on this criterion, sera from 62.79%, 60.47% and 72.09% of donors were positive for anti-SaCas9, anti-AsCas12a and anti-CasΦ antibodies respectively. Immunoblotting was used as an orthogonal method to evaluate seropositivity to Cas proteins in the donor samples. The detection of anti-Cas antibodies in sera from individual donors is depicted in Fig. 2b. The percentage of donors whose sera tested positive for anti-SaCas9, anti-AsCas12a, and anti-CasΦ antibodies by ELISA and immunoblotting is shown in Fig. 2c.

### Pre-existing T cell responses to Cas proteins

**Fig. 3:**
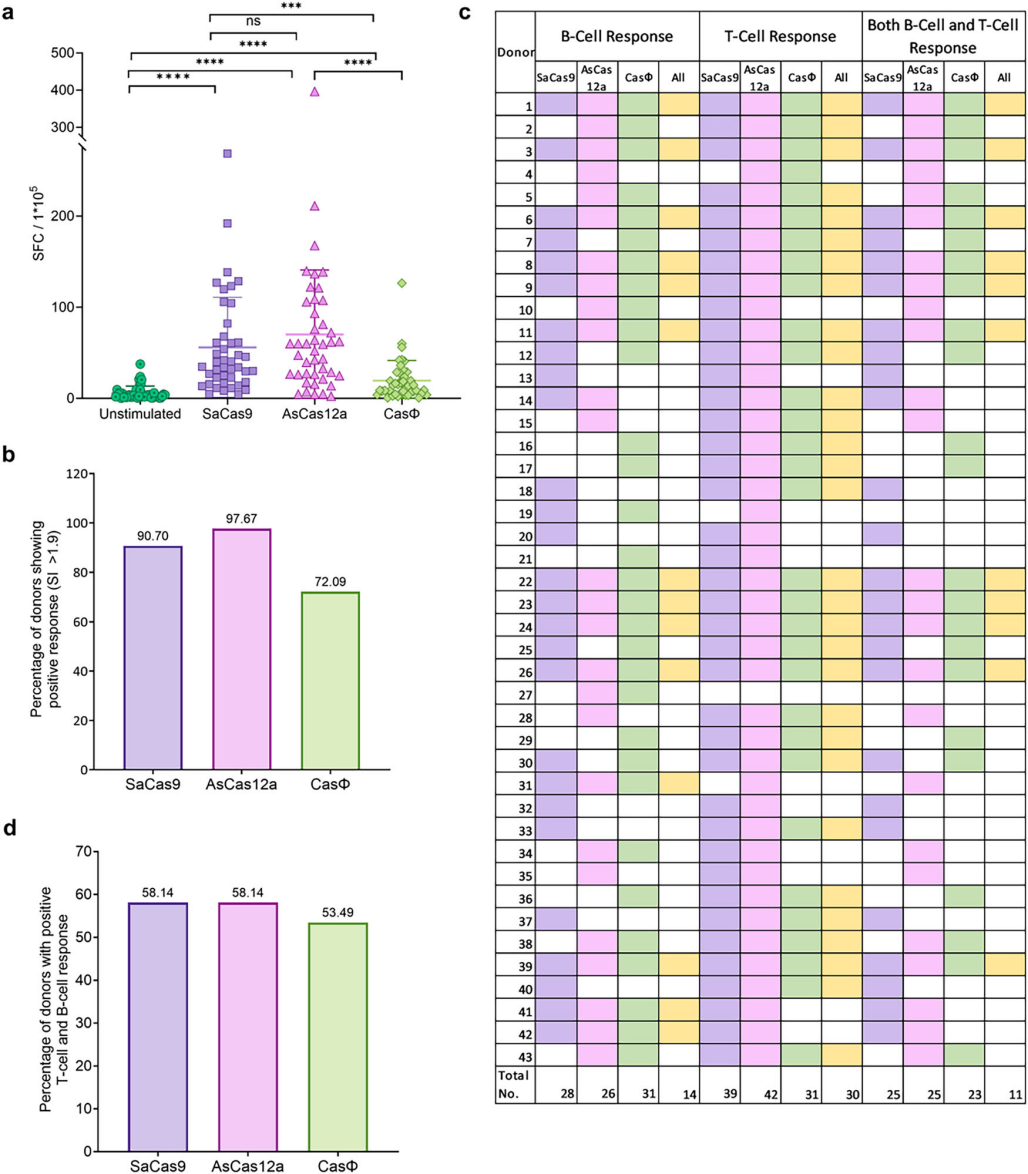
Detection of T cell response to different Cas proteins. **a** IFN-γ ELISpot assay results showing Spot Forming Cells (SFC)/ 1*10^5^. PBMCs from 43 healthy donors were stimulated with different Cas proteins to detect IFN-γ secreting cells. Unstimulated PBMCs were used as control. Treatment with all Cas proteins resulted in significantly higher SFCs compared to the unstimulated controls. Statistical significance was analyzed using two-tailed paired student t-test and *P*-values were determined. *P ≤ 0.05; **P ≤ 0.01; ***P ≤ 0.001; ****P ≤ 0.0001, ns – non-significant. Data is presented as mean ± SD. Each point represents one independent donor (*n* = 43 biological replicates). Technical replicate measurements were averaged prior to statistical analysis. Detailed statistics are provided in the Suppl. Table 4. **b** Percentage of donors showing positive response to the Cas proteins. A donor was considered positive when the Stimulation Index (S.I.) was > 1.9. **c** Individual donor response plot of positive B cell, T cell, and both B and T cell response for each Cas protein. Donors that responded to all three Cas proteins are shown in yellow. **d** Percentage of the donors showing both B and T cell responses for the three Cas proteins. Source data are provided as a Source Data file.

We also determined whether the 3 Cas proteins used in this study elicited antigen-reactive T cells using the interferon-γ (IFN-γ) enzyme-linked immunospot (ELISpot) assay. The ELISpot assay was carried out using peripheral blood mononuclear cells (PBMCs) derived from the same 43 donors whose sera were used to detect anti-Cas antibodies. Antigens (SaCas9, AsCas12a, and CasΦ) were incubated with the PBMCs and the IFN-γ released from the antigen-reactive T cells were determined by ELISpot. Consistent with previous studies^21,33,34^, the number of spots forming cells (SFCs) associated with IFN-γ release were significantly (*P* < 0.0001) higher for SaCas9-treated cells compared to the untreated controls (Fig. 3a). We obtained similar results for PBMCs treated with AsCas12a and CasΦ which resulted in significantly (*P* < 0.0001) higher numbers of SFCs associated with IFN-γ secretion compared to the untreated controls (Fig. 3a). There were no significant differences between numbers of SFCs observed when PMBCs were treated with SaCas9 or AsCas12a. However, there was a significant decrease in the number of SFCs in PBMCs treated with CasФ compared to PMBCs treated with either SaCas9 or AsCas12a.

To estimate the percentage of donors that showed a positive response to each of the Cas proteins we calculated the stimulation index (SI) (see Methods for details). A donor was considered positive when the SI was > 1.9. Based on this criterion, 90.70%, 97.67%, and 72.09% of donors had pre-existing T cell response to SaCas9, AsCas12a, and CasΦ respectively (Fig. 3b).

### Comparing B and T cell responses of individual donors

Figure 3c illustrates the B and T cell response of individual donors based on the cut-off criteria we used (see above). More donors showed a memory T cell response than active anti-Cas antibodies in the sera. Nonetheless, over 50% of the donors (58.14%, 58.14% and 53.49% for SaCas9, AsCas12a and CasΦ respectively) showed the presence of anti-Cas antibodies as well as a positive T cell response (Fig. 3d).

### Transduction of Cas proteins into mono-allelic cell lines

MHC Associated Peptide Proteomics (MAPPs) is a powerful tool to identify peptides derived from a specific antigen presented by MHC proteins on antigen-presenting cells^35,36^. We have previously refined this technology for the identification of peptides derived from therapeutic proteins (including Cas9) isolated from MHC-II proteins^22,37^. The rationale for identifying peptides bound to MHC-II molecules is that most therapeutic proteins such as antibodies or replacement proteins are injected as purified proteins^38^. Such endogenous antigens are ingested into endocytic compartments of macrophages, dendritic cells or B cells are presented to CD4^+^ T cells as peptides bound to MHC II molecules^29^. The approach, however, may not be fit-for-purpose while estimating immunogenicity risk when a modality (such as a Cas proteins) is delivered as a gene and is synthesized in vivo by the patient’s cells. Endogenously synthesized antigens in the cytosol of all cells are presented to CD8^+^ T cells as peptides bound to MHC I molecules^29^.

To allow the identification of Cas-associated peptides on MHC-I molecules we developed a novel workflow that is depicted in Supplementary Fig. 3. For this assay we used 9 monoallelic cell lines wherein each cell line was engineered to express a single MHC-I allele. The 9 MHC-I variants are: A*02:01, A*11:01, A*23:01, A*24:02, B*07:02, B*08:01, B*27:02, B*35:01 and B*44:02 and the generation of these cell lines has been described previously^39,40^. Our overarching strategy was to express the 3 Cas genes in these cells lines and identify the MHC-I associated proteins using mass spectrometry.

The monoallelic cells were transduced with the Cas genes (see Methods for details). The transduced cells (i.e., GFP^+^/HLA-A, B, C^+^) were separated using a cell sorter and expanded. The process was repeated multiple times. Dot plots showing the identification of cells positive for both MHC-I and the Cas proteins are depicted in Supplementary Fig. 4a. Using this strategy, we generated a total of 27 cell lines transduced with SaCas9, AsCas12a and CasΦ.

In immunoblots, the monoallelic cell lines transduced with SaCas9, AsCas12a and CasΦ showed bands corresponding to ~130 kDa, ~160 kDa and ~76 kDa respectively (Supplementary Fig. 4b-d which are consistent with the size of these proteins.

### Identification of Cas-derived peptides bound to MHC-I proteins

The transduced monoallelic cell lines SaCas9, AsCas12a, and CasΦ were used to identify Cas-derived peptides processed and presented by the MHC-I complexes using an MHC-Associated Peptide Proteomics (MAPPs) assay (see Methods). The SaCas9, AsCas12a and CasΦ derived peptides in each of the 27 cell lines were identified after HLA class-I based affinity purification and MS analysis. Measured spectra were analyzed using Fragpipe database search peptide identification (using a false discovery rate of 1%). The MHC Motif Decon analysis allowed confident attribution of the majority of identified peptides to the expressed monoallelic HLA class I molecules (on average 94% of the identified peptides per sample (min 86%- max 99%)) (Suppl. Fig. 5a, b). The peptide length distribution and histograms analysis per sample are shown in (Suppl. Figs. 6−9)^41^.

**Fig. 4:**
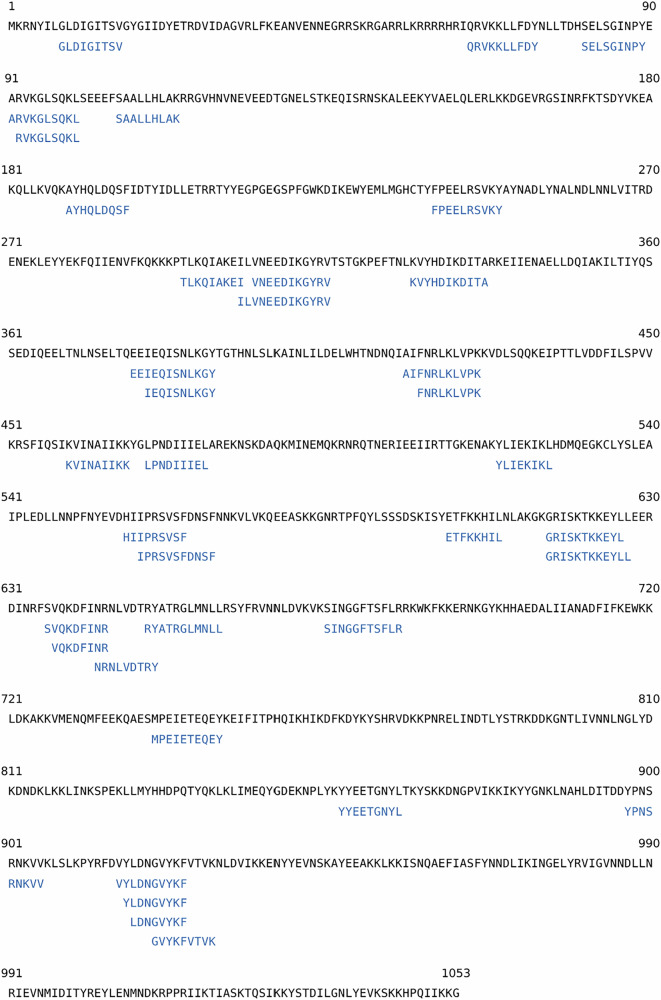
Alignment of SaCas9-derived peptide sequences identified from MAPPs Assay with the full-length amino acid sequence of SaCas9 protein.

**Fig. 5:**
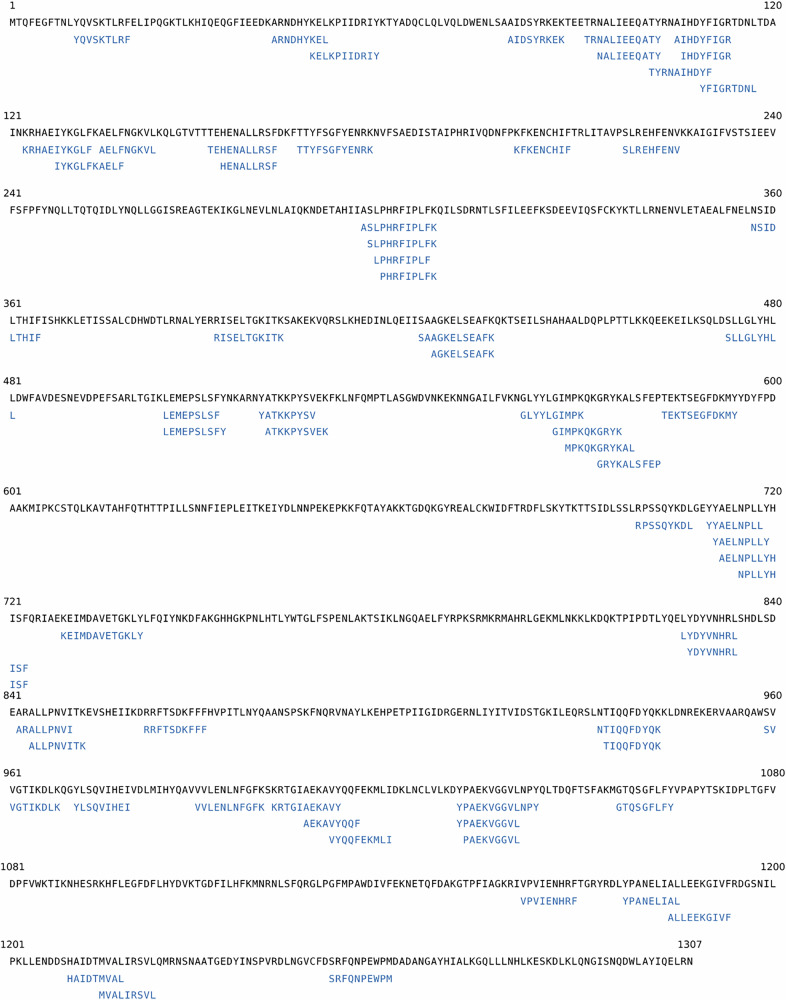
Alignment of AsCas12a-derived peptide sequences identified from MAPPs Assay with the full-length amino acid sequence of AsCas 12a protein.

**Fig. 6:**
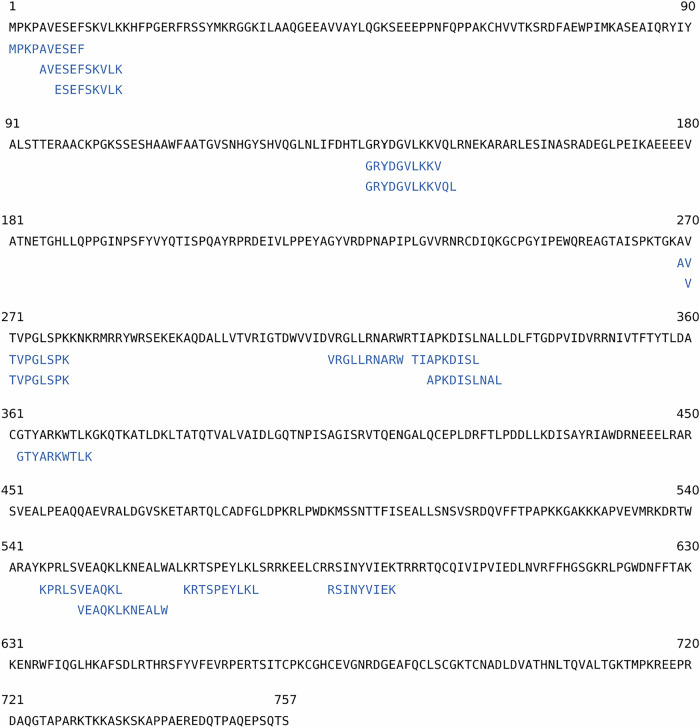
Alignment of CasΦ-derived peptide sequences identified from MAPPs Assay with the full-length amino acid sequence of CasΦ protein.

For each of the monoallelic cell lines, the Cas-derived peptides identified are shown in Table 1. The positions of the Cas-derived peptides on the amino acid sequences of SaCas9, AsCas12a, and CasΦ are depicted in Figs. 4, 5, and 6 respectively.

**Table 1:** Cas-derived peptides identified on MHC-I molecules expressed on the surface of monoallelic cell lines

		A*02:01	A*11:01	A*23:01	A*24:02	B*07:02	B*08:01	B*27:02	B*35:01	B*44:02
SaCas9	Total number of peptides	8034	7692	4845	5634	5579	4253	5196	10530	8240
	Total number of Cas-peptides	4	8	3	6	4	5	5	3	3
	% Cas-peptides	0.049	0.104	0.062	0.106	0.072	0.118	0.096	0.028	0.036
AsCas12a	Total number of peptides	9660	10534	6575	6090	7337	6830	8021	9165	9760
	Total number of Cas-peptides	4	19	7	7	4	3	9	9	10
	% Cas-peptides	0.041	0.180	0.106	0.115	0.055	0.044	0.112	0.098	0.102
CasΦ	Total number of peptides	10856	14959	9089	9277	10456	8685	9816	12278	12825
	Total number of Cas-peptides	1	6	1	0	3	1	4	2	1
	% Cas-peptides	0.009	0.040	0.011	0	0.029	0.012	0.041	0.016	0.008

### Functional evaluation of a SaCas9 peptide identified in the MAPPs assay

**Fig. 7:**
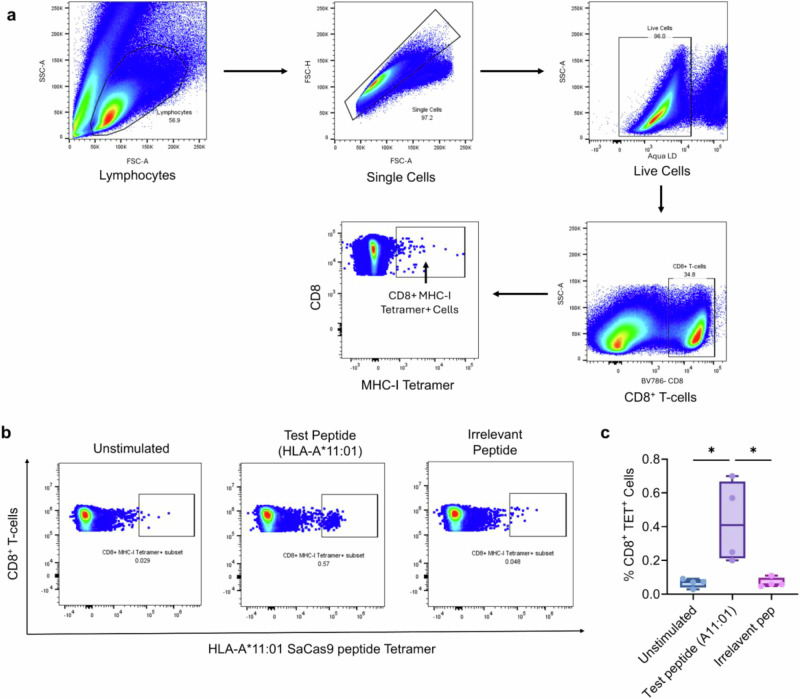
Expansion of HLA-A*11:01-restricted SaCas9-specific CD8^+^ T cells following MAPPs-derived peptide stimulation. **a** Representative gating strategy for identification of antigen-specific CD8^+^ T cells. Sequential gating was performed on lymphocytes, singlets, live cells, CD8^+^ T cell subset followed by detection of tetramer-positive CD8^+^ T cells using APC-labelled HLA-A*11:01 peptide GVYKFVTVK tetramer. **b** Representative donor showing 20-fold increase in CD8^+^ MHC-I tetramer^+^ cells in the PBMCs expanded with the peptide GVYKFVTVK compared to unstimulated control whereas the irrelevant peptide only resulted in a 1.7-fold increase. **c** Plot showing the percentage of CD8^+^ MHC-I tetramer^+^ cells in 4 donors. PBMCs expanded with the GVYKFVTVK test peptide resulted in a significant increase in the CD8^+^ MHC-I tetramer^+^ cells compared to the unstimulated and irrelevant peptide controls. Box plots show the median (center line), the 25th and 75th percentiles (box limits), and the minimum and maximum values (whiskers). Each point represents one independent donor (*n* = 4 biological replicates). Statistical significance was analyzed using one-way ANOVA with Tukey’s multiple comparisons and the *P*-values are reported- Unstimulated vs. test peptide (A11:01), *P = 0.0141; Unstimulated vs. irrelevant peptide ns, *P* = 0.9997; Test peptide (A11:01) vs. irrelevant peptide, *P = 0.0145. F (2, 9) = 8.645. Source data are provided as a Source Data file.

We carried out a proof-of-principle assessment of the relationship between MAPPs data and functional T cell responses. We selected the SaCas9 peptide GVYKFVTVK identified in the MAPPs assay on SaCas9-transduced monoallelic cells expressing HLA-A*11:01. Our aim was to determine whether this peptide could elicit antigen-specific CD8⁺ T cell responses in donors who carry the HLA-A*11:01 allele. We performed ex vivo stimulation assays using PBMCs from HLA-A*11:01-positive donors. Monocyte-derived dendritic cells (moDCs) were generated, matured, and pulsed with either the HLA-A*11:01-restricted peptide or an irrelevant control peptide prior to co-culture with autologous PBMCs under cytokine-supported expansion conditions. Following two rounds of peptide stimulation over 20 days, cells were harvested and analyzed by flow cytometry using an APC-conjugated HLA-A*11:01 tetramer. Antigen-specific CD8⁺ T cells were identified through sequential gating of lymphocytes, singlets, viable cells, and CD8⁺ subsets (Fig. 7a).

Stimulation with the GVYKFVTVK peptide identified in the MAPPs assay resulted in a significantly (*P* = 0.0141) higher number of CD8^+^ cells that were tetramer positive compared to unstimulated controls. In contrast, stimulation with an irrelevant control peptide did not result in a significantly (*P* = 0.9997) higher number of CD8^+^ cells that were tetramer positive (Fig. 7b). The irrelevant peptide is a peptide derived from SaCas9 that was identified in the MAPPs assay for the allele HLA-B*08:01 (YLIEKIKLH). These results were obtained using PBMCs from four independent donors (Fig. 7c) which all carried the HLA-A*11:01 allele. The second HLA-A alleles in these donors were HLA-A*30:01, -A*24:02, and -A*68:03. Thus, none of these donors carried the HLA-B*08:01 allele that is associated with the irrelevant peptide. These findings demonstrate that the MAPPs-derived peptide efficiently expands SaCas9-specific CD8⁺ T cells ex vivo in an HLA-A*11:01-restricted context, indicating its capacity to drive antigen-specific cellular immune responses.

### Structural mapping of Cas-derived peptides identified in MAPPs assays

**Fig. 8:**
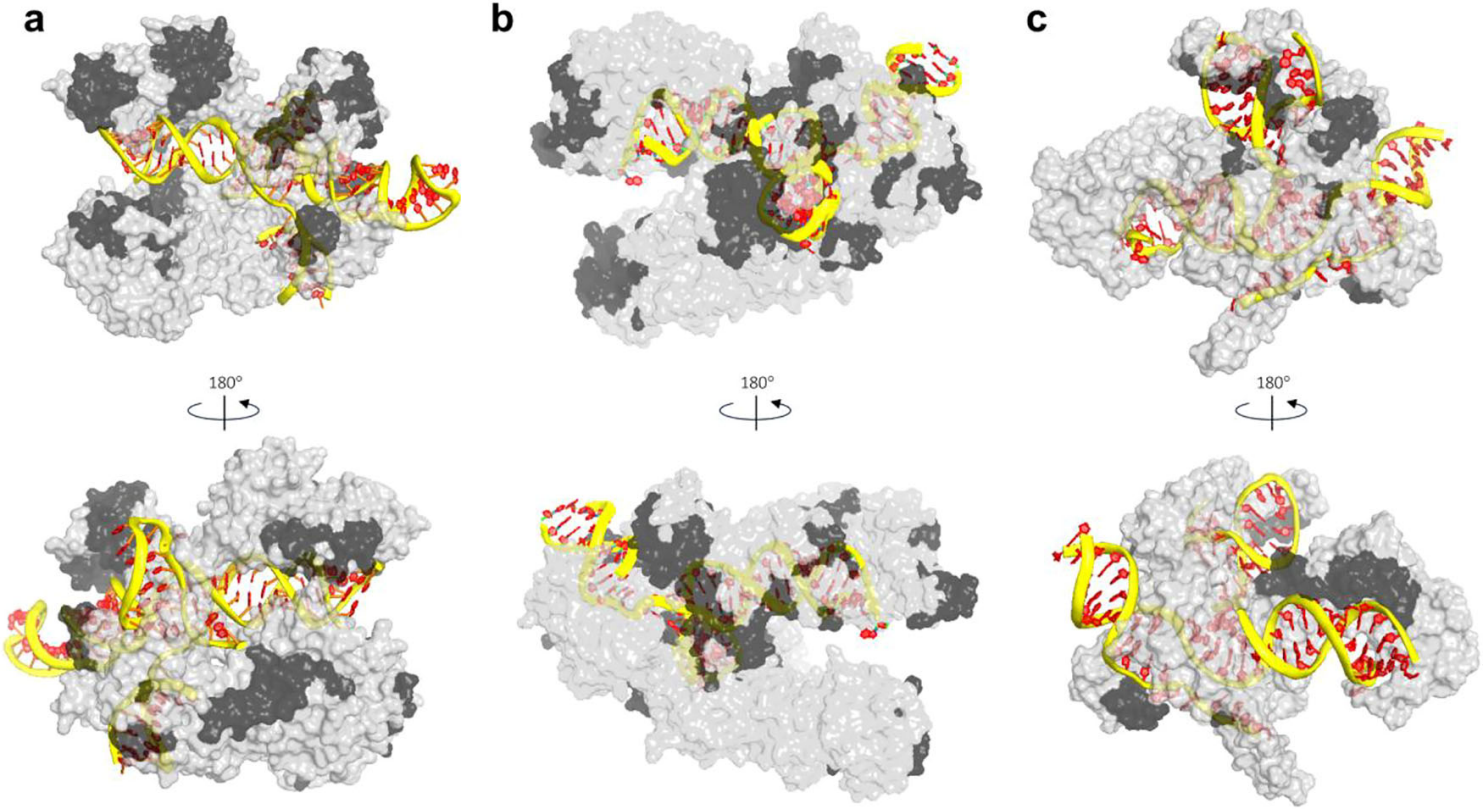
Structural analyses. **a** X-Ray structures of SaCas9 in complex with sgRNA and target DNA (PDB: 5CZZ). **b** AsCas12a in complex with crRNA and DNA (PDB: 5B43). **c** CasΦ in complex with crRNA and DNA (PDB: 7LYS). Sequences identified in the MAPPs assay (black) were superimposed on the structures of each of the Cas proteins (grey).

**Fig. 9:**
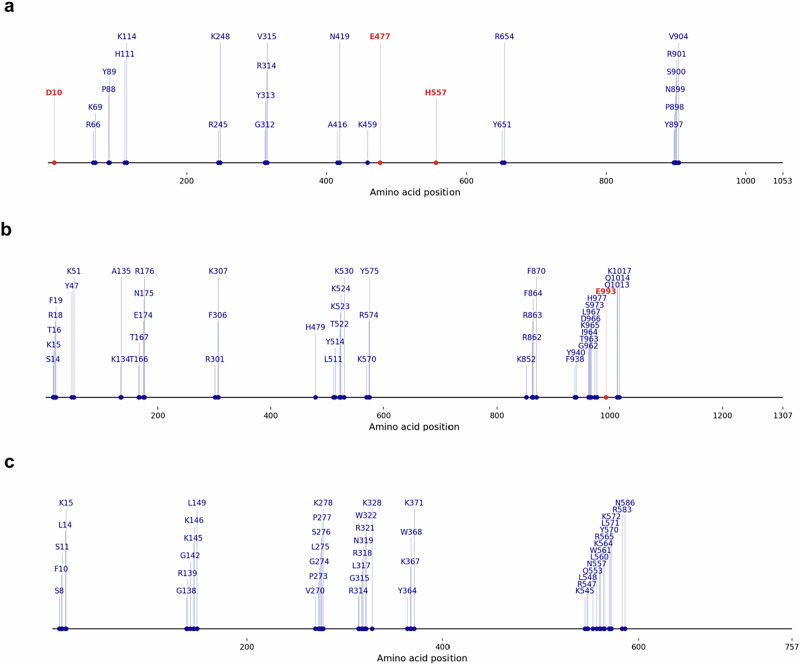
Structural analyses and overlap of residues. **a** SaCas9 residues corresponding to peptide sequences identified in the MAPPs assay that overlap with nucleic-acid contact residues (blue) and catalytic sites (red). **b** AsCas12a residues corresponding to peptide sequences identified in the MAPPs assay that overlap with nucleic-acid contact residues (blue) and catalytic sites (red). **c** CasΦ residues corresponding to peptide sequences identified in the MAPPs assay that overlap with nucleic-acid contact residues (blue).

We identified 36, 65, and 15 unique peptides from SaCas9, AsCas12a, and CasΦ respectively. It is important to determine which of these peptides align with regions of their respective Cas proteins that are important for gene editing. Peptides identified from each of the 3 Cas proteins in the MAPPs assay were mapped onto available high-resolution structures of SaCas9 (Fig. 8a), AsCas12a (Fig. 8b), and CasΦ (Fig. 8c). Residues corresponding to peptide sequences were examined for spatial overlap with nucleic-acid contact residues and catalytic sites. In SaCas9, 23 peptide-mapped residues overlap with nucleic-acid contact residues (Fig. 9a), and peptide coverage includes the catalytic residues D10 and E477 within the RuvC nuclease domain and H557 within the HNH domain, which together form the catalytic centers responsible for cleavage of the non-target and target DNA strands, respectively. In AsCas12a, 45 peptide-mapped residues overlap with nucleic-acid contact residues (Fig. 9b), and peptide coverage includes E993, a catalytic residue within the RuvC active site that is required for DNA cleavage. In CasΦ 44 peptide-mapped residues overlap with nucleic-acid contact surfaces (Fig. 9c), but none coincide with residues forming the catalytic center of the RuvC domain. Residues overlapping nucleic-acid contacts are positioned along the nucleic-acid binding channel where they contribute to electrostatic and backbone interactions with the RNA guide or DNA target. Consequently, substitutions at these sites would be expected to influence nucleic-acid recognition or duplex stabilization, whereas mutations affecting catalytic residues would impair nuclease activity.

Importantly, the identification of peptide-mapped residues that overlap with nucleic-acid–interacting surfaces but are spatially separated from catalytic residues provides a structural framework for rational protein engineering aimed at reducing immunogenic epitopes while preserving nuclease activity. Residues located on solvent-exposed regions that overlap predicted immunogenic peptides but are distal from catalytic centers represent candidates for targeted substitution designed to disrupt antibody or T cell recognition without perturbing the structural integrity of the nuclease or its nucleic-acid binding interface.

## Discussion

Studies on immune responses to Cas9 proteins have demonstrated preexisting antibodies^21,22,28^ and memory T cell responses^20,21,28^. An additional study also identified promiscuous T cell epitopes^42^. These findings are not surprising because the Cas9 proteins, SaCas9 and Sp-Cas9, are derived from human pathogens^43^. It has however, been speculated that Cas proteins from other sources may be less immunogenic, or at the very least, not exhibit pre-exiting immunity in human populations^27^. Here, we provide a comparative evaluation of immunogenicity risk for three Cas proteins (SaCas9, AsCas12a, and CasΦ).

The natural sources of the three Cas proteins described here are: a human pathogen (SaCas9)^21^, a human commensal (AsCas12a)^44^ and a “biggie” (or jumbo) phage (CasΦ)^27^. Our results show that sera from 62.79, 60.47, and 72.09 percent of donors test positive for anti-SaCas9, AsCas12a, and CasΦ antibodies respectively. Moreover, 46.5, 53.5, and 60.5 percent of donors test positive in two orthogonal assays, ELISA and an immunoblot. We used an in-silico tool^32^, to ensure that the cohort of 43 donors used in this study has a distribution of MHC-I and MHC-II variants that is representative of the North American population. The MHC proteins play a central role in determining whether an individual elicits an immune response to a protein therapy^29^. The MHC proteins are also the most polymorphic in the human genome^31^. By ensuring that the cohort used in this study is representative of our population of interest, it is reasonable to assume that the frequencies of preexisting antibodies to the three Cas proteins could reflect those in the general population. Moreover, PBMCs from the same cohort elicited T cell responses in 90.70, 97.67, and 72.09 percent of donors when challenged with SaCas9, AsCas12a, and CasΦ proteins respectively. Taken together these data show that a substantive proportion of the North American population has pre-existing antibodies and a memory T cell response to SaCas9, AsCas12a, and CasΦ. We did not observe significant differences between SaCas9, AsCas12a, and CasΦ with respect to levels of pre-existing antibodies in the donor cohort. However, T cell responses (SFC/10^5^ PBMCs in an ELISpot assay) were significantly lower (*P* ≤ 0.001) for CasΦ compared to SaCas9 or AsCas12a.

As discussed above B and T cell responses to SaCas9 have been reported previously and expected. The AsCas12a gene editor has been isolated from a human commensal^44^. Previous studies on commensals have shown that gut bacteria are targeted by IgA antibodies, which primarily occur in the mucosa^45^. Studies have also identified systemic commensal-targeting IgG antibodies in human and mouse blood^46^. Thus, our data is consistent with current understanding of the immune responses to commensals. However, while immune responses to commensals are expected, it does not necessarily follow that antibodies to a specific protein isolated from a gut bacterium will be identified in the blood. Our identification of antibodies that specifically target AsCas12a is thus important from a drug development perspective.

The identification of pre-existing antibodies and a memory T cell response to CasΦ is more difficult to explain. CasΦ is a family of Cas proteins encoded in the Biggiephage clade^27,47^. These phages have only recently been discovered^27^ and there is limited information about their hosts, distribution in the ecosystem etc. However, multiple studies have demonstrated phage-specific humoral immune responses^48–50^. Human exposure to and preexisting immunity to phage-derived proteins is therefore plausible. Additional studies of interactions between humans and bacterial species that are host to CasΦ are warranted.

Contrary to previous speculations^27^, we demonstrate pre-existing immune responses to Cas proteins other than the human pathogen-derived Cas9. Immunogenicity risk assessment however, demands that we consider not only the probability of an immune response but also the consequences in the context of the specific medical intervention^51,52^. In many applications, Cas-mediated gene editing will be carried out following a “one and done” approach. Thus, unlike life-long protein infusions for chronic diseases or even multiple infusions of antibodies for human cancers, gene editing is expected to result in a cure, and additional treatments may not be needed^2,3,7^. There is, of course, no long-term clinical experience with in vivo gene editing but one animal study does provide some insights^53^. In this study, the in vivo consequences of pre-existing immunity to SaCas9 were evaluated in the context of liver genome editing with AAV packaged CRISPR-Cas9 in a mouse model. Efficient genome editing occurred in mouse liver with pre-existing SaCas9 immunity. This suggests that even if a patient were to have pre-existing antibodies and/or a memory that results in the rapid generation of antibodies, the desired clinical outcome would not be affected. However, genome editing was followed by an increase in CD8^+^ T cells in the liver and a cytotoxic T cell response. These immune responses led to hepatocyte apoptosis, loss of recombinant AAV genomes, and complete elimination of genome-edited cells. In another study, AAV-CRISPR therapy in dystrophic canine models initially restored dystrophin but triggered strong Cas9-specific humoral and CD8^+^ T cell responses, leading to muscle inflammation and loss of therapeutic benefit. These immune responses were not mitigated by promoter restriction or transient immunosuppression, highlighting Cas9 immunity as a major barrier^54^. These results imply that, for Cas-based therapeutics which are delivered via viral vectors, an MHC-I mediated activation of CD8^+^ cytotoxic T cells pose a larger risk than an MHC-II mediated humoral response. The data also shows that the activation of CD8^+^ T cells is likely to affect both the efficacy of gene editing and elicit strong safety signals that could be life-threatening.

The only clinical report of in vivo CRISPR therapy describes baby KJ with CPS1 deficiency. The therapy showed early clinical improvement and reduced disease burden. However, these findings are limited to a single patient with short-term follow-up, and important questions remain regarding long-term safety, durability of editing, immune responses, and broader clinical applicability^55^. To understand and circumvent the MHC-I mediated cytotoxic T cell response of the 3 Cas proteins it is important to identify the MHC-I associated T cell epitopes^56,57^. When delivered intracellularly, an antigen is processed by antigen-presenting cells and presented through the MHC-I molecules to CD8^+^ T cells^31^. In the context of therapeutic protein products, the MAPPs assay has been increasingly used to measure antigen processing and presentation as a part of the T cell immune response^22,35,37^. However, identification of peptides associated with MHC-I proteins requires a completely different workflow that includes the intracellular expression of the gene of interest. In developing this workflow, we used 9 monoallelic cell lines, wherein each cell line expressed a single MHC-I allele^39,40^. The expression of all 3 Cas proteins was verified in these cell lines, and Cas-derived peptides identified. We identified 36, 65, and 15 unique SaCas9, AsCas12a, and CasΦ-derived peptides presented on the 9 MHC-I variants. In our previous study, almost all (85%) of SaCas9 peptides identified on MHC-II variants induced T cell proliferation. It is not surprising that most peptides presented on antigen presenting cells initiated a T cell response because SaCas9 is a protein of bacterial origin, i.e., almost completely foreign to human immune systems. Thus, almost all the peptides presented by antigen presenting cells are likely be recognized by T cell receptors. Thus, it is likely that many of the Cas-derived peptides identified on the MHC-I proteins are also likely to elicit CD8^+^ T cell responses. To demonstrate antigen-specific CD8^+^ responses by specific peptides is challenging. However, using a peptide that was identified on the monoallelic expressing the HLA-A*11:01 allele, we demonstrated that the MAPPs-derived peptide efficiently expands antigen-specific CD8⁺ T cells using a tetramer-based assay.

The identification of epitopes is important because these can provide specific targets for deimmunization. Across 9 MHC-I alleles, we identified 25, 36, and 9 peptide regions from SaCas9, AsCas12a, and CasΦ respectively. For the most part, peptides identified using the MAPPs assay represent only a fraction of Cas-derived peptides that bind with high affinity ( < 5^th^ percentile) to each of the MHC-I variants (Table 2). The most common approaches to deimmunization involve protein engineering to decrease peptide-MHC binding affinities^35,37,58,59^. Although we have identified a relatively small set of peptides as T cell epitopes, multiple epitopes can contribute to T cell activation, making it unlikely that the immune response can be fully engineered around, in contrast to scenarios involving a single dominant epitope^60^. Consequently, the engineering of sufficient peptides to reduce the binding affinity for multiple MHC-I variants while simultaneously preserving gene editing functions and limiting off-target effects remains a formidable challenge. In addition to protein engineering approaches, alternative delivery strategies may help mitigate immunogenicity risks. For example, delivery of Cas9 as a ribonucleoprotein (RNP) complex limits the duration of intracellular exposure, which may reduce antigen presentation and subsequent immune activation^61^. The structure-based analyses of T cell epitopes, provided here, suggest a plausible avenue for structure-guided epitope modification strategies. 36, 65, and 15 epitopes were identified on SaCas9, AsCas12a, and CasΦ, respectively. However, the structural analysis shows that 3 epitopes on SaCas9, 1 epitope on AsCas12a, and none of the epitopes on CasΦ are in the catalytic sites. This suggests that most of the targets for engineering de-immunized Cas proteins are not directly engaged in the catalytic process of gene editing. It is nonetheless plausible that engineering these sites could have allosteric effects that affect gene-editing function or specificity.

**Table 2:** Comparing numbers of high affinity (percentile score < 5) Cas-derived peptides identified in silico using NetMHC and the numbers of peptides identified using the MAPPs assay

Allele	SaCas9			AsCas12a			CasΦ		
	NetMHC	MAPPs	% identified in MAPPs	NetMHC	MAPPs	% identified in MAPPs	NetMHC	MAPPs	% identified in MAPPs
HLA-A*02:01	82	4	4.88	118	4	3.39	48	1	2.08
HLA-A*11:01	107	8	7.48	147	19	12.93	70	6	8.57
HLA-A*23:01	87	3	3.45	162	7	4.32	49	1	2.04
HLA-A*24:02	91	6	6.59	171	7	4.09	49	0	0.00
HLA-B*07:02	71	4	5.63	117	4	3.42	69	3	4.35
HLA-B*08:01	144	5	3.47	198	3	1.52	72	1	1.39
HLA-B*27:02	98	5	5.10	107	9	8.41	78	4	5.13
HLA-B*35:01	104	3	2.88	163	9	5.52	83	2	2.41
HLA-B*44:02	128	3	2.34	136	10	7.35	69	1	1.45

There are several limitations to this study. The donor cohort (*N* = 43) is relatively small. We would note, however, that we used a previously developed algorithm^32^ to generate the donor cohort. The algorithm utilizes simulated annealing to optimizes a cohort to match the HLA distribution of the target population. This algorithm performed 7 standard deviations better than 10 million randomly picked cohorts. Consequently, our cohort, though modest in size, is meaningfully representative of the North American population with respect to MHC diversity. Secondly, our structure-based assessments of the epitopes that affect function (an important criteria for de-immunization) are based on an in silico analysis and were not empirically validated. A third, and perhaps more consequential, limitation concerns the relationship between the MAPPs data and functional T cell responses. The MAPPs assay identifies peptides that are processed and presented on MHC-I molecules; however, the identification of a peptide in the MAPPs assay does not, by itself, confirm that the peptide will elicit an antigen-specific CD8⁺ T cell response in vivo. Establishing this concordance between MHC-I-associated peptide presentation and functional cellular immunity is critical for interpreting the immunological significance of MAPPs-identified epitopes and for prioritizing candidates for deimmunization strategies. In the current study, we performed a proof-of-principle functional assessment using a single SaCas9-derived peptide (GVYKFVTVK) identified on monoallelic cells expressing HLA-A11:01. We demonstrated that this peptide drives significant antigen-specific expansion of CD8⁺ T cells in HLA-A11:01-positive donors using a tetramer-based assay. While these findings are encouraging and support the biological validity of our MAPPs workflow, they represent only a limited, single-peptide assessment across one HLA allele and one Cas protein. The 116 unique peptides identified across SaCas9, AsCas12a, and CasΦ in the current study (spanning 9 MHC-I alleles) have not been functionally validated. This leaves open the important question of what fraction of MAPPs-identified peptides from each Cas protein can elicit functional CD8⁺ T cell responses, and whether the rate of concordance is consistent across different HLA alleles and Cas protein origins. Assays that can be routinely used to assess the functional importance of peptides identified in MAPPs assays represent an unmet need. Such studies are currently ongoing. These future efforts will be critical for establishing the predictive value of the MAPPs-based MHC-I workflow described here and for translating the epitope map into actionable deimmunization targets.

Taken together, our data suggest that the immunogenicity-risk associated with the 3 Cas proteins, SaCas9, AsCas12a, and CasΦ, is high. Currently, there is insufficient evidence to make predictions of clinical immunity based on in vitro and ex vivo assays. Nonetheless, the science-based immunogenicity risk assessments described here could help to begin defining the immunogenicity risk of Cas proteins and drive the selection of the most appropriate Cas protein-delivery system for individual applications. When more clinical experience is gained with Cas-based therapies the experimental tools we have described can be refined to better predict the impact of immunogenicity on clinical safety and efficacy.

## Method

### Ethics approval and informed consent

PBMCs were obtained from healthy volunteer donors enrolled in the National Institutes of Health (NIH) protocol “Collection and Distribution of Blood Components from Healthy Donors for In Vitro Research Use” (IRB protocol 99CC0168). The protocol was approved by the NIH Institutional Review Board. Written informed consent was obtained from all participants prior to enrollment and sample collection; no waiver of informed consent was granted. Samples were de-identified before distribution to investigators for research use.

### HLA typed Peripheral blood mononuclear cells (PBMCs)

PBMCs were isolated from leukapheresis products (leukopaks) by density-gradient centrifugation using Ficoll-Paque PLUS (Cytiva, catalog number: 17544202) according to the manufacturer’s instructions. Following isolation, cells were washed twice with phosphate-buffered saline (PBS) (Gibco, catalog number: 20012050), and viability was assessed by trypan blue exclusion. Cells were then cryopreserved in fetal bovine serum (FBS) (Gibco, catalog number: 16140071) containing 10% dimethyl sulfoxide (DMSO) (ThermoFisher Scientific, catalog number: BP231-100) and stored in the vapor phase of liquid nitrogen using a controlled rate freezing protocol until use^33^. Donor demographic information, including age, sex, and HLA genotype, is summarized in Suppl. Table 2. High-resolution HLA typing was performed using sequence-based typing methods. HLA class I loci (HLA-A, HLA-B, and HLA-C) and, where applicable, HLA class II loci (HLA-DRB1), were determined and used for donor selection through the in-house algorithm “SampPick”^32^. The serum samples from the same cohort of donors were used to screen for pre-existing Cas antibodies.

### Calculation of allelic frequency

To calculate the North American allele frequency in our donor cohort, we used allele frequencies data base (http://www.allelefrequencies.net/default.asp) which contains all alleles and the corresponding allele frequencies of several populations. Once we obtained the frequencies to specific alleles (as of 29th April 2025), we normalized the data using following the equation (1) to compare the frequencies between North American population and our experimental cohort.

$$\left\{\frac{{Frequency}\,{of}\,{the}\,{allele}}{{Sum}\,{of}\,{the}\,{frequecy}\,{of}\,{all}\,{alleles}}\right\}*100\,$$

### Assessment of recombinant protein purity and endotoxin levels

SaCas9 and CasΦ proteins were expressed in *Escherichia coli* (*E. coli*) and purified to > 90% purity (Genescript, NJ). The proteins were endotoxin-free and were formulated in 50 mM Tris-HCl, 500 mM NaCl, and 10% glycerol (pH 8.0) for SaCas9 and in 50 mM Tris-HCl, 500 mM NaCl, and 10% glycerol (pH 8.0) for CasΦ. AsCas12a protein was provided by Editas medicine^44^, the protein was formulated in 20 mM Tris HCL, 300 mM NaCl, 0.1 mM EDTA, 50% Glycerol, 1 mM DTT (pH 7.4). Purified recombinant SaCas9, AsCas12a, and CasΦ proteins were analyzed by SDS–PAGE to assess purity and apparent molecular weight. Protein samples (1 µg per lane) were prepared in denaturing loading buffer (ThermoFisher Scientific, catalog number: NP0008), heat-denatured, and resolved on polyacrylamide gels alongside a pre-stained protein ladder (ThermoFisher Scientific, catalog number: LC5800). Following electrophoresis, gels were stained and imaged under identical acquisition settings. The bands corresponding to the molecular weights for each protein were imaged and reported in Suppl. Fig. 1a.

Endotoxin levels were quantified using an endpoint chromogenic Limulus Amebocyte Lysate (LAL) assay (ThermoFisher Scientific, catalog number: A39552) according to the manufacturer’s instructions. Briefly, 50 μL of LAL enzyme reagent was added to 50 μL of endotoxin standards or protein samples in a 96-well flat-bottom plate and gently mixed. Following incubation at 37 °C for 10 min, 100 μL of pre-warmed chromogenic substrate was added, and the reaction was incubated for an additional 6 min at 37 °C. The reaction was terminated by adding 100 μL of 25% acetic acid (ThermoFisher Scientific, catalog number: A38-500) to each well. Absorbance was measured at 405 nm using a Victor X3 plate reader (PerkinElmer). Endotoxin concentrations were calculated from a standard curve generated by linear regression and reported as endotoxin units per milligram of protein (EU/mg) in Supplementary Fig. 1b.

### ELISA for detection of anti-Cas antibodies

96-well ELISA plates (ThermoFisher Scientific, catalog number: 3455) were coated with 100 µL per well of either SaCas9, AsCas12a, or CasΦ (concentration, 1 µg/mL) in 1X carbonate-bicarbonate buffer (Sigma-Aldrich, catalog number: C3041-100CAP) overnight at 4 °C. Plates were washed three times with TBS with Tween (TBST, pH 8.5) (Sigma, catalog number: T9039-10PAK) and blocked with 200 µL per well of 1% BSA (ThermoFisher Scientific Blocker BSA, catalog number: 37520) in 1X TBS (Fisher Bioreagents, catalog number: BP24711). The plates were incubated for 1 h at room temperature (RT) and then washed three times with 200 µL per well of TBST. Healthy donor human serum was diluted (1:50) in blocking buffer, and 100 µL was added per well, and the plates were incubated for 1 h at RT. Plates were then washed with TBST as described previously. HRP-conjugated goat anti-human IgG Fc secondary antibody (Novus, catalog number: NB7449) was diluted to 1:100,000 in 1% BSA blocking buffer, and 100 µl was added per well. The plates were incubated for 1 h at RT and then washed three times with TBST. 100 µL per well of 3,3′,5,5′-Tetramethylbenzidine substrate (TMB) solution (ThermoFisher Scientific TMB-ELISA substrate solution, catalog number: N301) was then added and allowed to develop for 15 min protected from light. The reaction was stopped with 1 N sulfuric acid, and the optical density (OD_450 nm_) was measured using BioTek Synergy HTX Multimode plate reader from Agilent Technologies. The mean OD values of the blank were subtracted from the actual values. Human serum albumin (HSA) was used at the negative control. To determine the baseline, the mean value of HSA + 3 SD was used. Serum dilutions ranging from 1:10, 1:20, 1:30, 1:40, 1:50, 1:75, and 1:100 were tested, and the EC50 values were reported.

### Western Blot for detection of anti-Cas antibodies

The SaCas9, AsCas12a, and CasΦ antigen were diluted in reducing sample buffer (Invitrogen, catalog numbers: NP0009 and NP0008) at a concentration of 0.1 mg/mL, and after boiling for 5 min at 95 °C, samples (2 µg/well) were loaded on 4–12% Bis-Tris polyacrylamide gels (Invitrogen, catalog number: NP0322BOX) and electrophoresed. Following SDS-PAGE, the gels were transferred to nitrocellulose membrane (catalog number: 170-4156, Bio-Rad, Hercules, CA) using a semidry transfer method. The membranes were blocked with 5% non-fat milk in TBST at for 1 h, then incubated overnight with serum samples (1:10 dilution) at 4 °C. The membranes were then washed for with TBST (5x) and incubated with HRP conjugated anti-human secondary antibody (catalog number: ab97175, Abcam, Cambridge, MA) at a dilution of 1:10000 for one hour at RT, washed (3x) with TBST and developed with SuperSignal™ West Pico PLUS Chemiluminescent Substrate (catalog number: 34580, ThermoFisher Scientific, Waltham, MA).

### In vitro antigenic stimulation of PBMCs with Cas proteins

Cryopreserved PBMCs from healthy donors were thawed, washed two times with RPMI medium (Gibco, catalog number: 11-875-093) containing anti-aggregate wash (CTL, catalog number: CTL-AA-005) at 330 g for 10 minutes. The cells were then resuspended in CTL Test medium (CTL, catalog number: CTLT-005) supplemented with 1% L-glutamine (Gibco, catalog number: 35050-061) and 10 µL/mL CD28/CD49d co-stimulatory antibody (BD Biosciences, catalog number: 347690) and counted to determine cell number and viability. Cells (1.5 × 10^6^ per well) were plated in a in 24-well plate and stimulated with 10 µg/mL SaCas9, AsCas12a or CasΦ protein. Staphylococcal Enterotoxin B (SEB), 1 ng/mL (Sigma, catalog number: S4881) was used as a positive control. Unstimulated samples treated with PBS in CTL Test media were used as negative controls. Sample plates were incubated at 37 °C, 5% CO_2_. On day 4, half of the cell culture media was replaced with fresh CTL Test medium supplemented with 1% L-glutamine, 10 µL/mL CD28/CD49d co-stimulatory antibody, and recombinant human IL-2 (Pepro Tech, catalog number: 200-02) at a concentration of 100 IU/mL and incubated at 37 °C, 5% CO_2_ for 48 hours. Cells were collected, counted, and used for re-stimulation.

### Re-stimulation and IFN-γ ELISPOT Assay

A total of 100,000 cells per well of pre-stimulated PBMCs (see previous section) were plated on pre-coated 96-well IFN-γ ELISPOT plates (CTL catalog number: hIFNgp-2M/5) in CTL test medium (100 µL per well) supplemented with 1% L-glutamine and co-stimulatory antibody. To avoid the saturation of spots in ELISPOT plate, we reduced the cells to 25,000 in SEB condition. Re-stimulation of PBMCs was carried out by adding Cas proteins (5 µg/mL). We added 0.5 ng/mL of SEB and PBS as positive and negative controls respectively. Plates were incubated for approximately 18 h at 37 °C in a 5% CO_2_ incubator the ELISPOT assay was performed per to the manufacturer’s instruction (CTL). CTL Immunospot SC Suite/Immunocapture analysis software was used to scan, count and quality control ELISPOT wells to read the spot-forming cells (SFC). To compare the percentage of donors showing positive response to each Cas protein, we calculated the stimulation index (SI) of the spots using the following equation (2):

$$	{Stimulation}\,{Index}\,\left({SI}\right)=\\ 	\frac{{Average}\,{Spot}\,{Forming}\,{Cells}\,\left({SFC}\right)\,{of}\,{each}\,{condition}}{{Average}\,{Spot}\,{Forming}\,{Cells}\,({SFC})\,{of}\,{unstimulated}\,{control}}$$

### Culture of mono-allelic cell lines

Nine cryopreserved monoallelic cell lines, each expressing a single MHC-I allele (A*02:01, A*11:01, A*23:01, A*24:02, B*07:02, B*08:01, B*27:02, B*35:01, and B*44:02)^39,40^ were thawed, washed two times with RPMI medium containing anti-aggregate wash by centrifugation at 1200 rpm for 5 minutes. The mono-allelic cells were grown in DMEM + GlutaMAX + high glucose + Na Pyruvate (Gibco, catalog number: 10569-010) media supplemented with 20% HI-FBS (Gibco, catalog number: 16140071), 1% Penicillin-Streptomycin (Gibco, catalog number: 15140122). The cells were passaged after 48 h, 2–3, and were used for lentivirus transduction.

### Lentivirus transduction of monoallelic cell lines

Passaged monoallelic cell lines were then collected, seeded in a V-bottom 96 well plate (Corning, catalog number: 3894) at a concentration of 25×10^3^ cells per well in triplicate and a single well with non-transduced control. The cells were then incubated for 4 h at 37 °C, 5% CO_2_. A few minutes before the completion of 4 h incubation, infection mix was prepared using DMEM+ high glucose + Na Pyruvate media supplemented with 20% FBS, Polybrene (Sigma-Aldrich, catalog number: TR-1003-G) at a working concentration of 8 µg/mL and target MOI (Multiplicity of infection) of 5 for purified lentivirus. The plate was centrifuged at 37 °C for 1 h at 1000 xg and incubated at 37 °C, 5% CO_2_ overnight. AsCas12a- and CasΦ-expressing lentiviral particles were obtained from GeneCopoeia (5′-CMV-SV40 NLS-AsCas12a-nucleoplasmin NLS-IRES2-eGFP, catalog number: LPP-CS-GS103N-Lv215-01-B00; 5′-CMV-SV40 NLS-CasΦ-nucleoplasmin NLS-IRES2-eGFP, catalog number: LPP-CS-GS102N-Lv215-01-B00). SaCas9-expressing lentiviral particles were produced similarly by GeneCopoeia and were difted by Editas Medicine (5’-CMV-SV40 NLS-SaCas9-nucleoplasmin NLS with IRES2-eGFP, catalog number: LPP-14105-Lv215-B00).

### Post-transduction culture and cell sorting

The transduced cells were transferred to a 48 well plate, supplemented with fresh DMEM + GlutaMAX+ high glucose + Na Pyruvate media (Gibco, catalog number: 10569-010) with 20% HI-FBS (Gibco, catalog number: 16140071), 1% Penicillin-Streptomycin (Gibco, catalog number: 15140122) and selected antibiotic for each cell line making the total volume to 1 mL and incubated at 37 °C, 5% CO_2_. After 48 h, the cells were transferred into a 6 well plate (Corning, catalog number: 3516) and supplemented with fresh media. The cells were expanded and sorted for GFP-positive and HLA-A, B, C APC-stained cells (APC anti-human HLA-A, B, C Antibody, BioLegend, catalog number: 311410) under sterile conditions using BD FACSAria Fusion sorter. The sorted cells were further expanded, and we performed a second cell sorting to enrich the double positive cells. The expanded cells were cryopreserved and stored in vapor liquid nitrogen until further use.

### Western blot analysis of Cas protein expression

The transduced monoallelic cells were lysed using the lysis buffer (ThermoFisher Scientific Pierce, catalog number: 1861603), PMSF (Phenylmethanesulfonyl fluoride solution) (Sigma, catalog number: 93482) and Halt Protease Inhibitor 100X (ThermoFisher Scientific catalog number: 1860932). The concentration of the protein was determined using BCA Assay kit (ThermoFisher Scientific catalog number: PI23225). Lysate was mixed with 4X LDS buffer (Invitrogen, catalog number: NP0008) and heated for 10 minutes at 85 °C. Approximately 90 µg SaCas9, or 60 µg of AsCas12a and CasΦ was loaded on a 4–12% gradient Bis-Tris Mini Protein gel (Invitrogen, catalog number: NP0322BOX), electrophoresed and transferred to a membrane using dry protein transfer. The membrane was blocked using 5% milk in TBST for 1 h and incubated with α-SaCas9 (polyclonal anti-body 1:1000, Editas Medicine)^19^, α-AsCas12a (monoclonal anti-body 1:1000, Editas Medicine)^44^ or α-CasΦ (monoclonal anti-body 1:1000, EnCor Biotechnology, catalog number: MCA-5F95) overnight at 4 °C. The membrane was then washed and incubated with a Goat anti-rabbit or anti-mouse HRP (1:10,000) secondary antibody (Abcam, catalog number:ab6721 and #ab6789) for 1 h at RT. After washing the membrane, SuperSignal West Pico PLUS chemiluminescent substrate (ThermoFisher Scientific, catalog number: 34580) was used to detect the protein in the membrane and imaged in iBright CL750 Imaging system (Invitrogen, catalog number: A44116).

### MAPPs

Frozen pellets of transduced monoallelic cells were lysed in mild lysis buffer (ImmuneSpec proprietary lysis buffer). Lysates were precleared on empty protein A Sepharose columns (Cytiva, Protein A Sepharose CL-4B, catalog number: 17096302) before affinity purification using monoclonal antibody clone W6/32 (IchorBio, catalog number: ICH1023) crosslinked to protein A-sepharose beads (Cytiva, Protein A Sepharose CL-4B, catalog number: 17096302). After mild acid elution the immunopeptides were selected by C18 solid phase extraction prior to analysis on a TimsTOF-SCP mass spectrometer: samples were loaded on Pure Evotips (Evosep Biosystems, catalog number: EV2013) and injected on an Evosep One nano LC system, which was equipped with a 150 mm × 150 µm Endurance column (30 samples per day gradient) and connected to a TimsTOF-SCP (Bruker Daltonics) mass spectrometer. The DDA PASEF method was employed (using an optimized Thunder-DDA-PASEF method, employing an HLA-I peptide tailored isolation polygon to select precursor ions for fragmentation) involving 1 TIMS-MS scan and 10 PASEF MS/MS scans. The TIMS-MS survey scan spanned 0.70 to 1.70 Vs/cm² with and 100–1,700 m/z, with a ramp time of 166 ms, while a collision energy ramp from 20 to 70 EV with an ion mobility range from 0.70 to 1.76 Vs/cm² was used for peptide fragmentation.

Raw MS/MS data were analyzed using MSFragger within the FragPipe pipeline (https://fragpipe.nesvilab.org/) (version 22.0). Database searches were performed against a custom FASTA file consisting of all reviewed human UniProt entries concatenated with the amino acid sequences of the Cas target proteins. Given the nature of MHC-bound peptides, searches were performed using unspecific cleavage settings, allowing identification of all peptides between 8 and 30 amino acids in length. Default fixed and variable modifications were applied, and peptide-spectrum matches were filtered using a 1% FDR at the peptide level.

### Peptide activation and flow cytometry analysis

Synthetic peptides corresponding to SaCas9-derived candidate epitopes and control antigens were obtained from a core facility and reconstituted at 1 mg/mL in 10% DMSO (ThermoFisher Scientific, catalog number: BP231-100). The amino acid (AA) sequences of the peptides were as follows: Test peptide – GVYKFVTVK (SaCas9 AA residues 921-929) and irrelevant peptide - YLIEKIKLH (SaCas9 AA residue 519-526). PBMCs from healthy donors were used to generate monocyte-derived dendritic cells (moDCs) by plastic adherence, followed by differentiation in IMDM (Gibco, catalog number: 12-440-061) supplemented with 10% fetal bovine serum (FBS), GM-CSF (50 ng/mL) (Peprotech, catalog number: 30003100UG), and IL-4 (20 ng /mL) (Peprotech, catalog number: 20004100UG). Media was refreshed on day 3 to maintain cytokine levels, and on day 5 cells were supplemented with TNF-α (50 ng /mL) (Peprotech, catalog number: 30001A100UG) to induce maturation. Mature DCs were harvested on day 7 and pulsed with synthetic peptides (20 μg/mL) for 2 h at 37 °C prior to co-culture with autologous PBMCs in CTL medium supplemented with GlutaMAX, CD28/CD49d, and IL-21 (30 ng/ mL) (Peprotech, catalog number: 200-21-100UG). During co-culture, 50% of the media was replaced every 3 days with fresh cytokine-supplemented medium containing IL-21. Fresh mature DCs were pulsed for 2 h with 10 µg/ml of peptides on day 14 in medium supplemented with IL-2 (10 ng /mL), IL-7 (5 ng /mL) (Peprotech, catalog number: 20007100UG), and IL-15 (25 ng/ml) (Peprotech, catalog number: 20012100UG). Previously stimulated and expanded PBMCs were collected and restimulated with freshly pulsed DCs and expanded until day 20. Subsequent media replenishment with IL-2, IL-7, and IL-15 was performed every 3 days to sustain T cell expansion.

On day 20, cells were harvested, washed, and prepared for flow cytometric analysis. Viability staining was performed using an amine-reactive live/dead dye (Invitrogen, catalog number: L34857), followed by Fc receptor blocking (Hu BD Fc Block, BD biosciences, catalog number: 564220). Cells were subsequently stained with CD8 (BD Horizon BV786 mouse anti-human CD8, BD Biosciences, catalog number: 563823) and MHC-I peptide-specific tetramer followed by acquisition on a FACS Aria-III cell sorter. Antigen-specific CD8⁺ T cells were identified using APC-conjugated MHC class I tetramers corresponding to the relevant HLA allele and peptide (e.g., HLA-A* 11:01), selected based on MAPPs data. Antigen-specific populations were identified through sequential gating of lymphocytes, singlets, live cells, CD8⁺ T cell subset and tetramer-positive cells.

### MHC Motif Decon tool analysis

To evaluate HLA-I peptide binding specificity and assign identified peptides to their most likely restricting allele, MHC Motif Decon (https://services.healthtech.dtu.dk/services/MHCMotifDecon-1.0/) was used^41^. For each monoallelic sample, the list of identified peptides was submitted together with the expressed HLA class I allele. The tool combines unsupervised clustering with binding predictions (NetMHCpan, default settings) to assign peptides to their most probable presenting HLA allele. Sequence logos were automatically generated for each peptide cluster to visualize the inferred HLA-I binding motif. The results enabled both QC assessment of allele specificity and downstream filtering of peptides based on predicted HLA-I restriction.

### Structural analysis

Structural analysis was performed using PyMOL (Schrödinger, LLC). Visualization of MAPPS-identified peptide-mapped residues was performed using Python. Residues identified by the MAPPS assay that overlap with nucleic acid (NA) contact sites or catalytic residues were compiled for SaCas9, AsCas12a, and CasΦ (Suppl. Fig. 7). For each variant, residues were classified into two categories: (i) those overlapping with nucleic acid contact sites (“MAPPS and NA overlap”) and (ii) those additionally coinciding with catalytic residues (“MAPPS and catalytic residue(s) overlap”). Lollipop plots were generated for each variant using a custom Python script (Python 3; matplotlib and openpyxl libraries).

### Data analysis

Statistical analyses were performed using GraphPad Prism 9.4.0 and Microsoft Excel. All graphics were made with GraphPad Prism 9.4.0. *p*-values ≤ 0.05 were considered statistically significant and significance was implied as follows: *P ≤ 0.05; **P ≤ 0.01; ***P ≤ 0.001; ****P ≤ 0.0001

### Reporting summary

Further information on research design is available in the Nature Portfolio Reporting Summary linked to this article.

## Supplementary information

Supplementary Information

Reporting Summary

Transparent Peer Review file

## Source data

Source Data

## Data Availability

All data are included in the Supplementary Information or available from the authors, as are unique reagents used in this Article. The raw numbers for charts and graphs are available in the Source Data file whenever possible. The MAPPs Mass Spectrometry data generated in this study have been deposited in the PRIDE Database under accession code: PXD073207.
