# Supplementary material for: Pre-existing antibody and T cell responses to SaCas9, AsCas12a and CasΦ are comparable in naïve individuals
Source: Nat Commun. 2026 Jul 1;17:8166. doi: 10.1038/s41467-026-75015-7 (PMC13457614; doi:10.1038/s41467-026-75015-7)
Supplement: Supplementary file 1 [file 41467_2026_75015_MOESM1_ESM.pdf]

Pre-existing Antibody and T cell Responses to SaCas9, AsCas12a and CasΦ Are Comparable in Naïve Individuals

Vijaya L. Simhadri[1], Pratima Bajgain[1], Sharon Vijayanand[1], Wojciech Jankowski[1], Atul Rawal[1], Lauren Thijs[2], Thomas Van Doninck[2], Kurt Boonen[2], Sofie Pattyn[3], Elise Pepermans[2*] and Zuben E. Sauna[1*]

[1]Division of Hemostasis, Office of Plasma Protein Therapeutics, Office of Therapeutic Products, Center for Biologics Evaluation and Research, Food and Drug Administration, Silver Spring, MD, USA. [2] ImmuneSpec, Galileilaan 18/6, 2845 Niel, Belgium. [3] IQVIA In vitro immunology, Rue Clement Ader 16, 6041 Charleroi, Belgium.

*Correspondence: zuben.sauna@fda.hhs.gov (Z.E. Sauna) or elise@immunespec.com (E. Pepermans)

Supplementary Figures

a

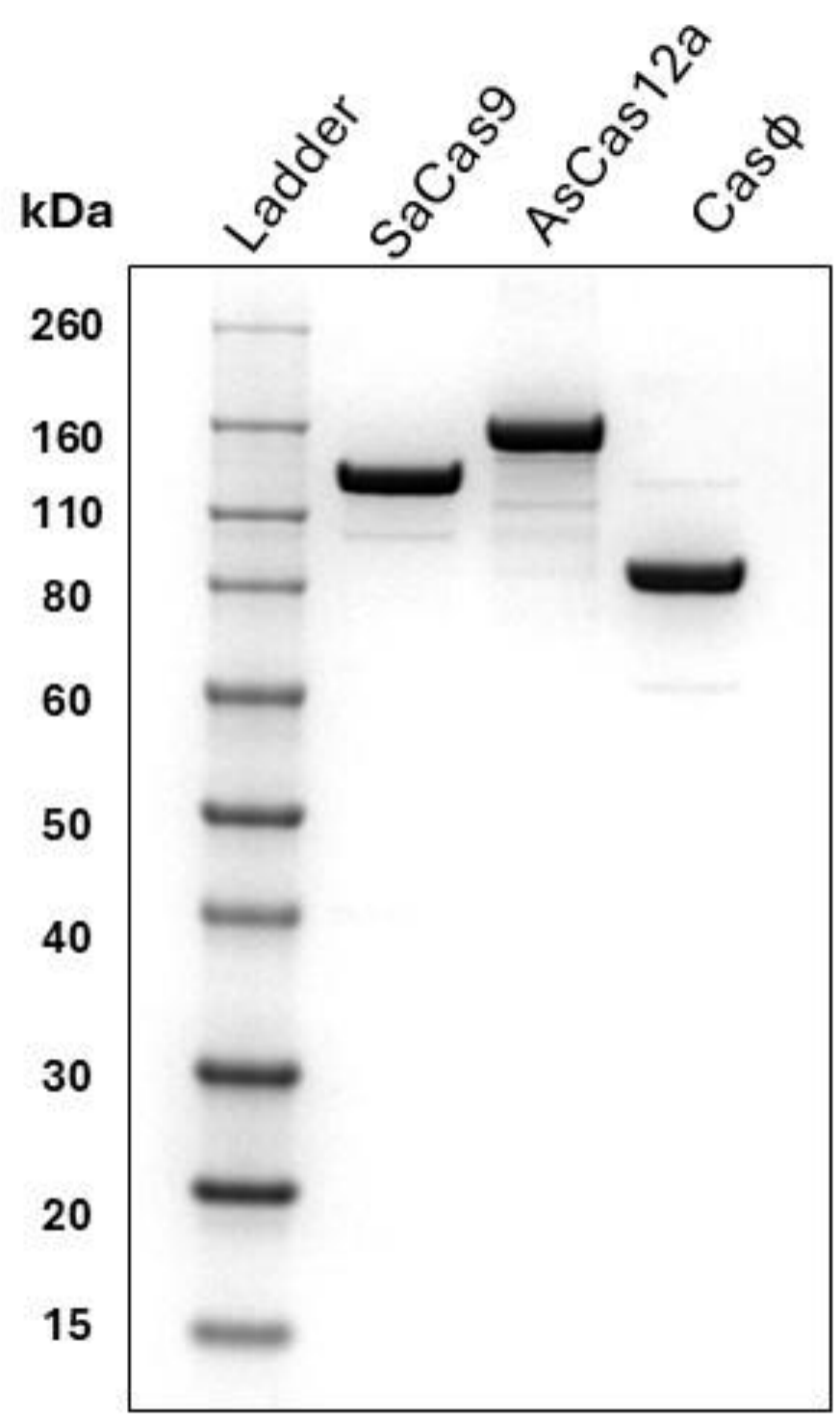

b

Protein	Endotoxin Level
SaCas9	0.2 EU/mg
Casϕ	≤0.3 EU/mg
AsCas12a	0.36 EU/mg

Supplementary Figure 1. Purity and endotoxin assessment of recombinant CRISPR proteins. a. SDS–PAGE analysis of purified SaCas9, AsCas12a and CasΦ proteins. A total of 1 µg of each protein was loaded per lane alongside a molecular weight ladder. Major bands corresponding to the expected molecular weights of SaCas9 (124–130 kDa), AsCas12a (151–153 kDa), and CasΦ (70–80 kDa) are observed, indicating high purity (n=2). b. Endotoxin levels of purified proteins measured using an endpoint chromogenic LAL assay. Endotoxin levels are reported as EU/mg, demonstrating low endotoxin contamination across all protein preparations (n=2).

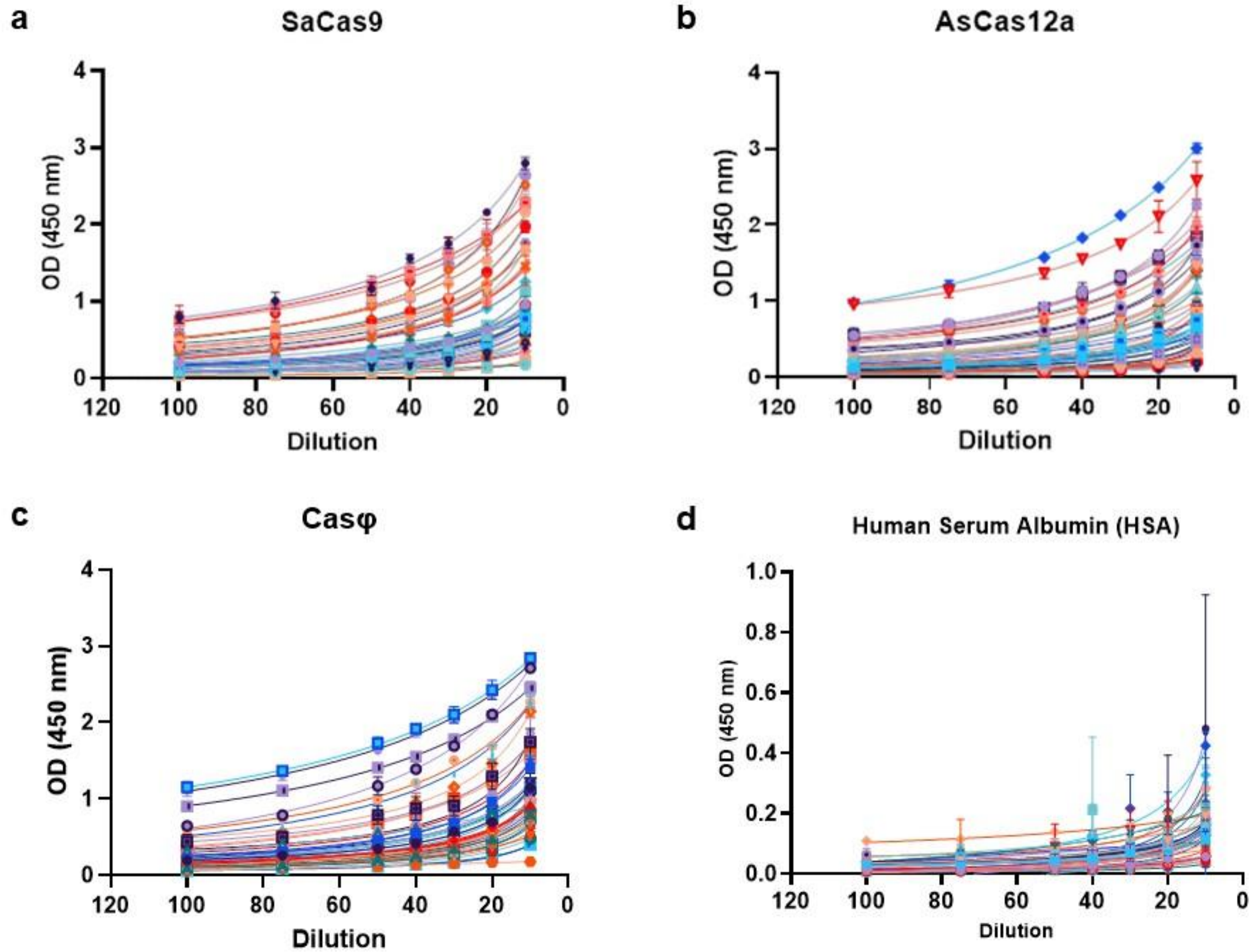

Supplementary Figure 2. ELISA serial titration curves (*n*=43 biological replicates). Serial titrations of 43 healthy donor serum ranging from 1:10, 1:20, 1:30, 1:40, 1:50, 1:75, and 1:100 were tested using ELISA for each Cas protein. The OD values were obtained and the blank values were subtracted. Data are presented as mean ± SD. Concentration–response curves were fitted by nonlinear regression using a three-parameter agonist response model and the EC50 values were calculated and reported in Supplementary Table 1. a. SaCas9. b. AsCas12a. c. CasΦ. and d. Human Serum Albumin (HSA) control.

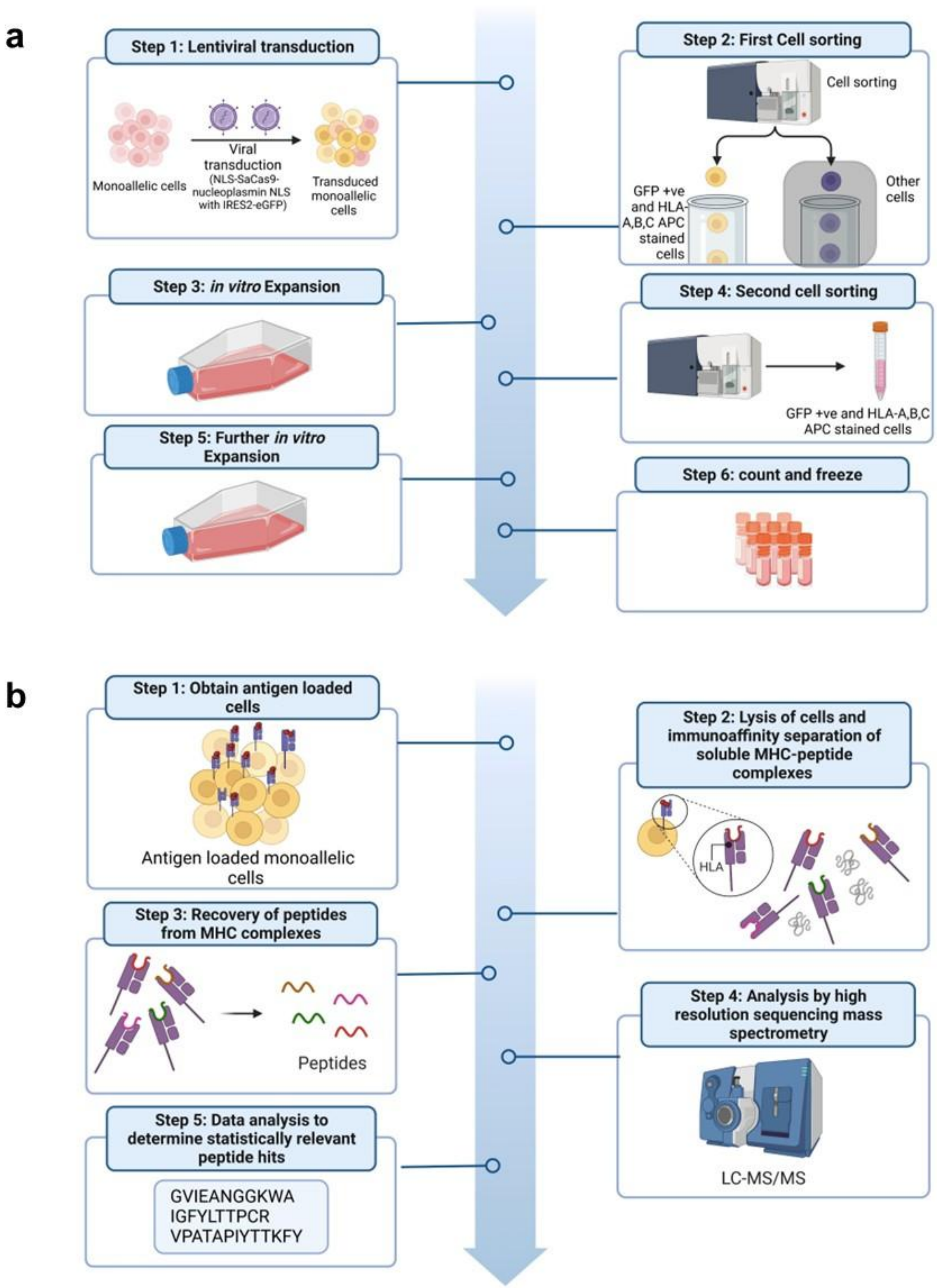

Supplementary Figure 3. Workflow of MHC Associated Peptide Proteomics (MAPPs) assay to identify Cas-associated peptides on MHC-I molecules. a. Lentiviral transduction and sorting of the monoallelic cells before performing MAPPs assay. b. MAPPs assay and identification of Cas-associated peptides. Created in BioRender. Simhadri, V. (2026) https://BioRender.com/wtsf6cr: Created in BioRender. Simhadri, V. (2026) https://BioRender.com/xaptg2b.

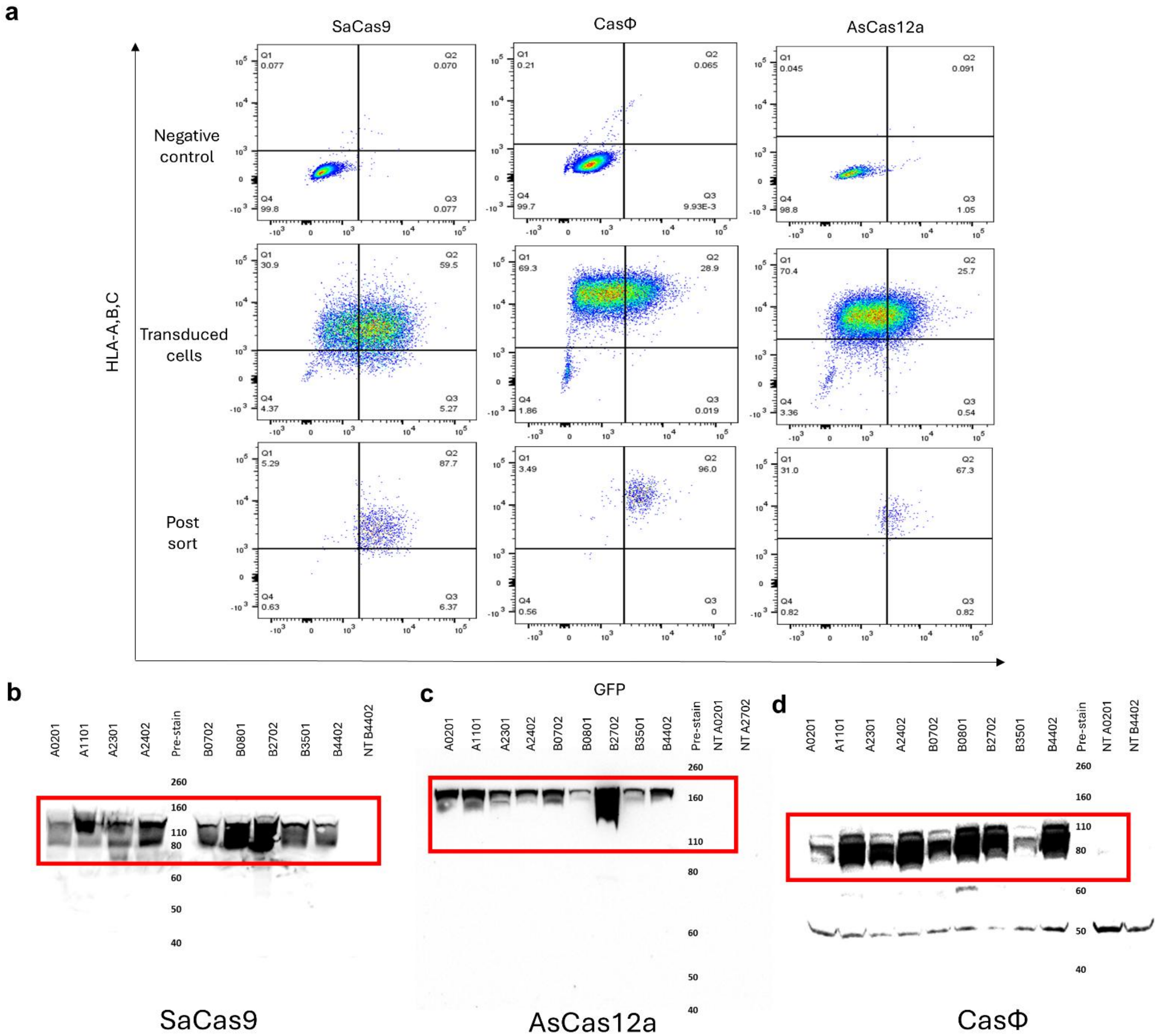

Supplementary Figure 4. Sorting of SaCas9, AsCas12a and CasΦ transduced monoallelic cells and verification of efficient lentivirus transduction of Cas proteins in monoallelic cells (n=2). a. Representative image showing the transduced monoallelic cells pre- and post-sorting. b. Immunoblot treated with rabbit α-SaCas9 antibody. A band around 130 kDa was observed for each transduced sample. Total protein amount = 25 µg. c. Immunoblot treated with mouse α-AsCas12a antibody. A band around 160 kDa was observed for each transduced sample. Total protein amount = 60 µg. d. Immunoblot treated with mouse α- Casϕ antibody. A band around 76 kDa was observed for each transduced sample. Total protein amount = 60 µg.

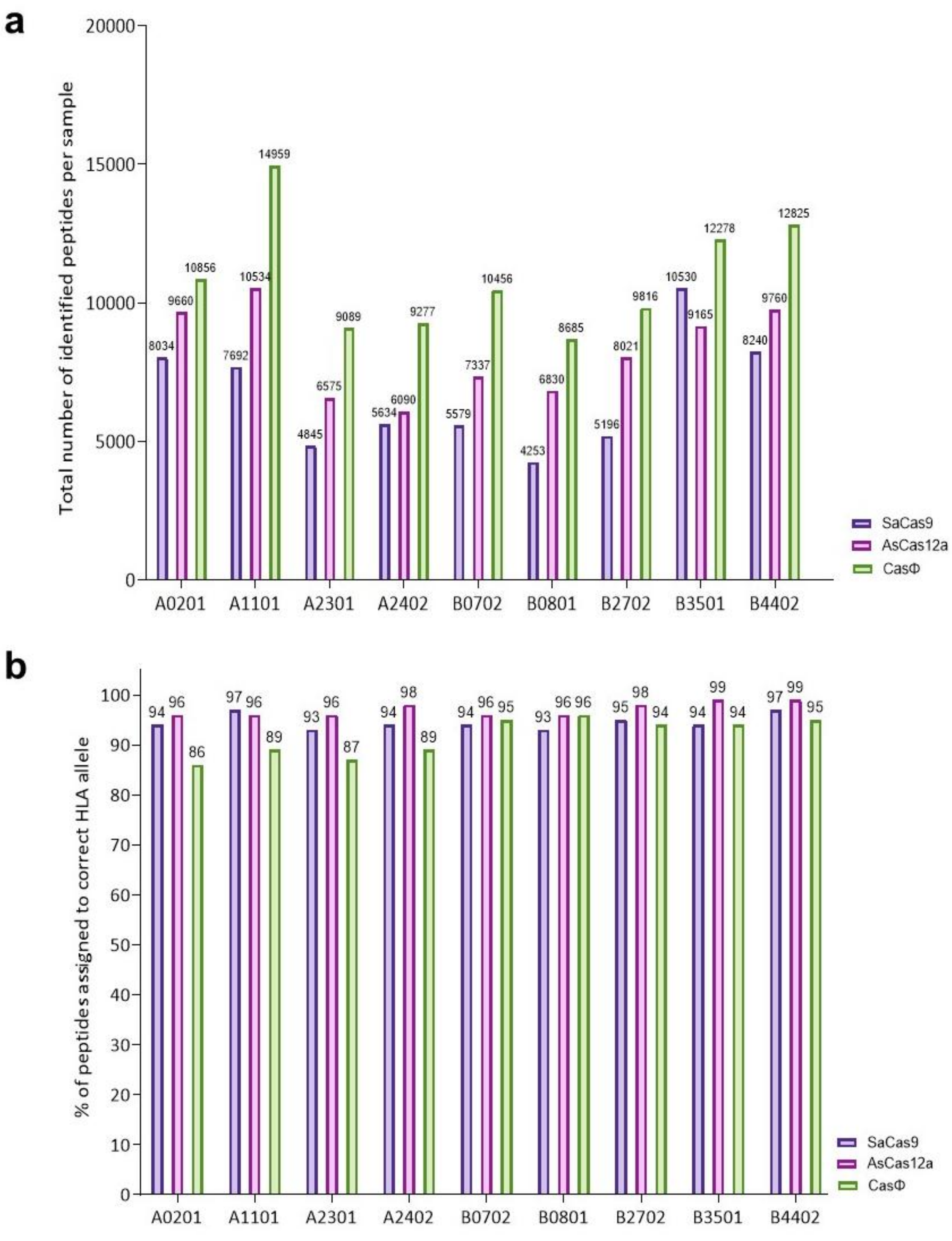

Supplementary Figure 5. Motif deconvolution of the HLA Class-I alleles. a. Total number of peptides identified from MAPPs assay for each monoallelic cell line transduced with SaCas9, AsCas12a and CasΦ. b. Percentage of identified peptides assigned to correct HLA Class-I alleles.

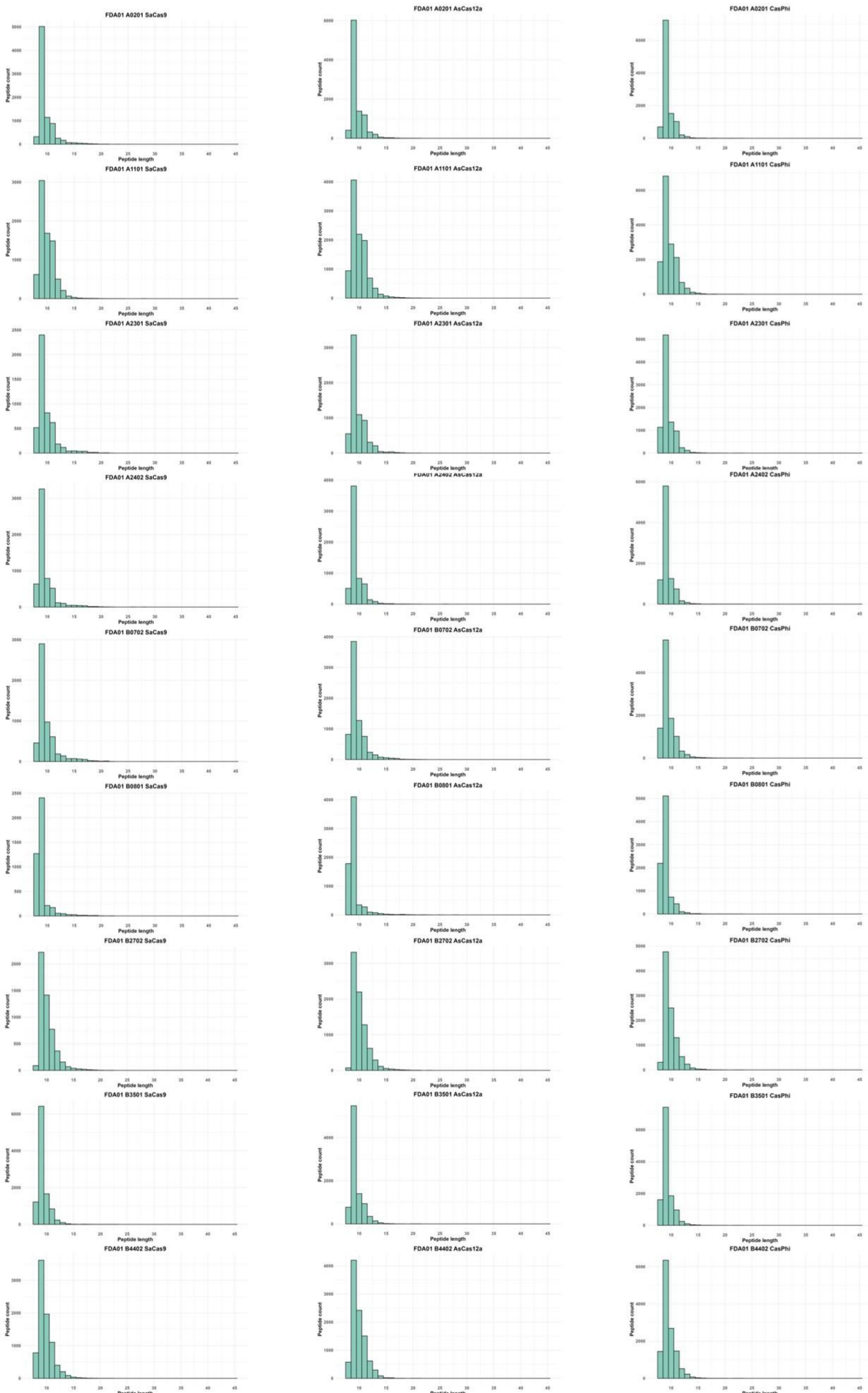

Supplementary Figure 6. Peptide length distribution per sample.

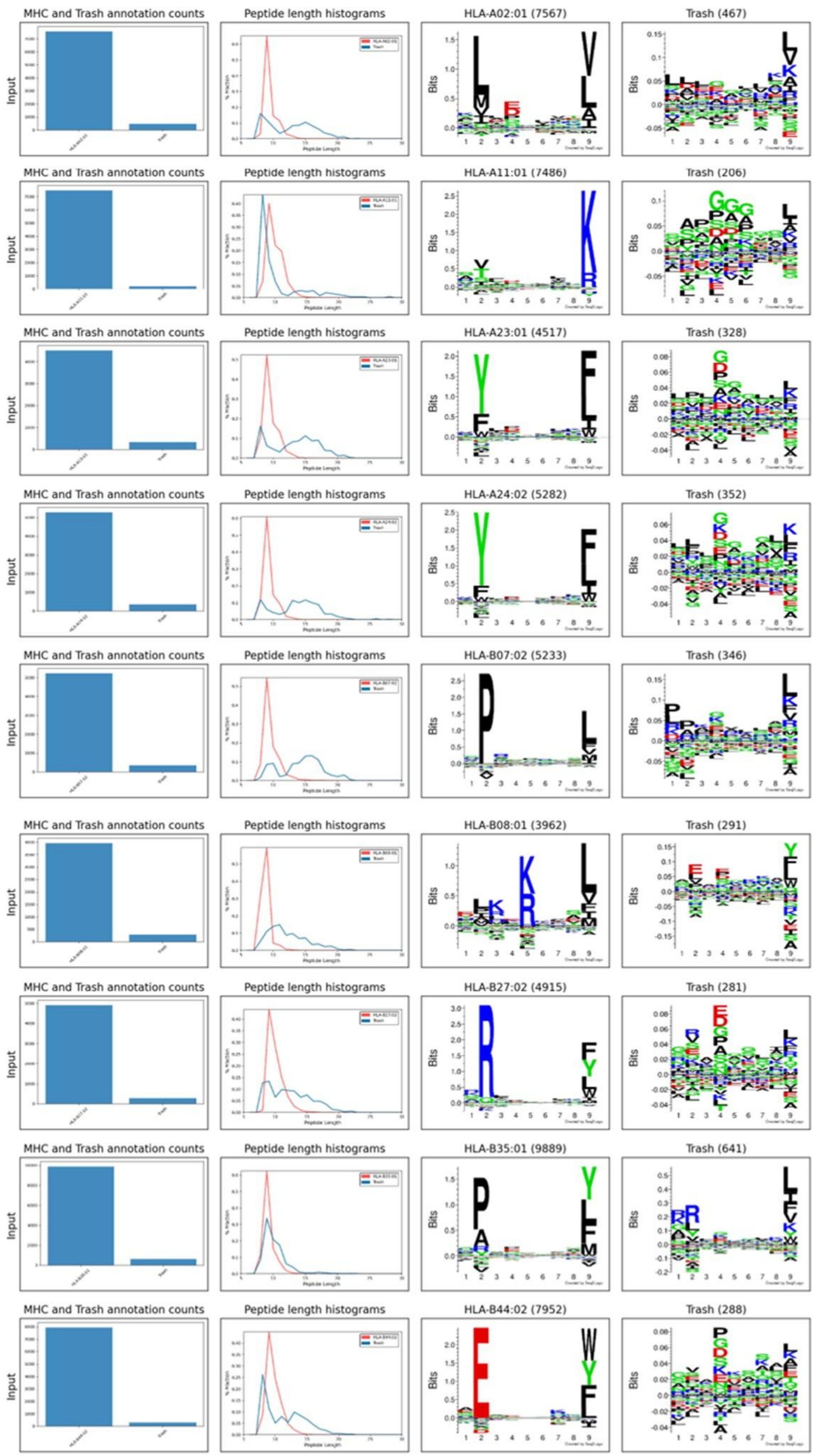

Supplementary Figure 7. SaCas9**.** Peptide length (6-30), excluding the use of the trash bin option. Peptides with a higher rank score than 20% are considered non-binder or contaminant and were assigned to the trash cluster.

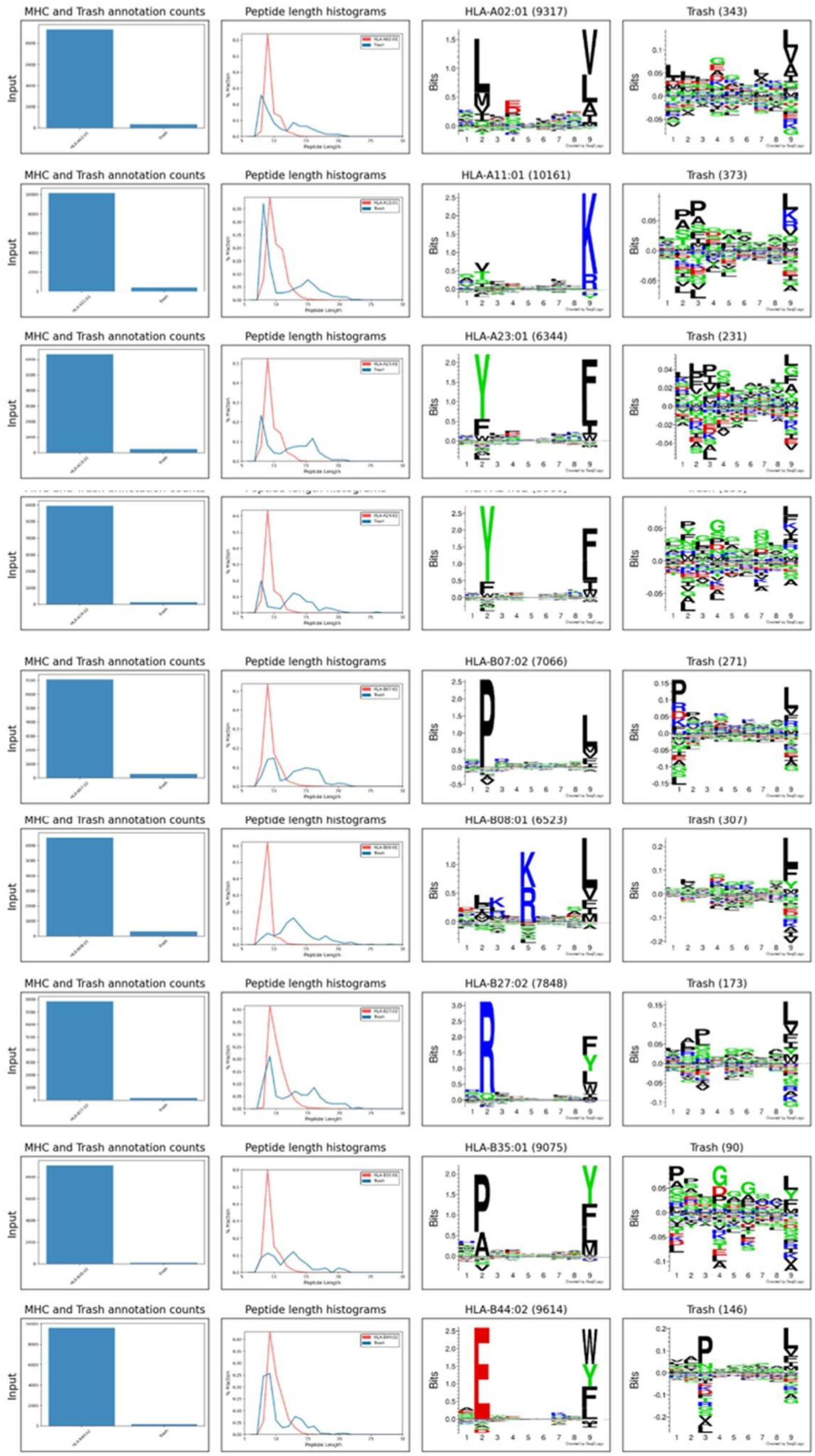

Supplementary Figure 8. AsCas12a. Peptide length (6-30), excluding the use of the trash bin option. Peptides with a higher rank score than 20% are considered non-binder or contaminant and were assigned to the trash cluster.

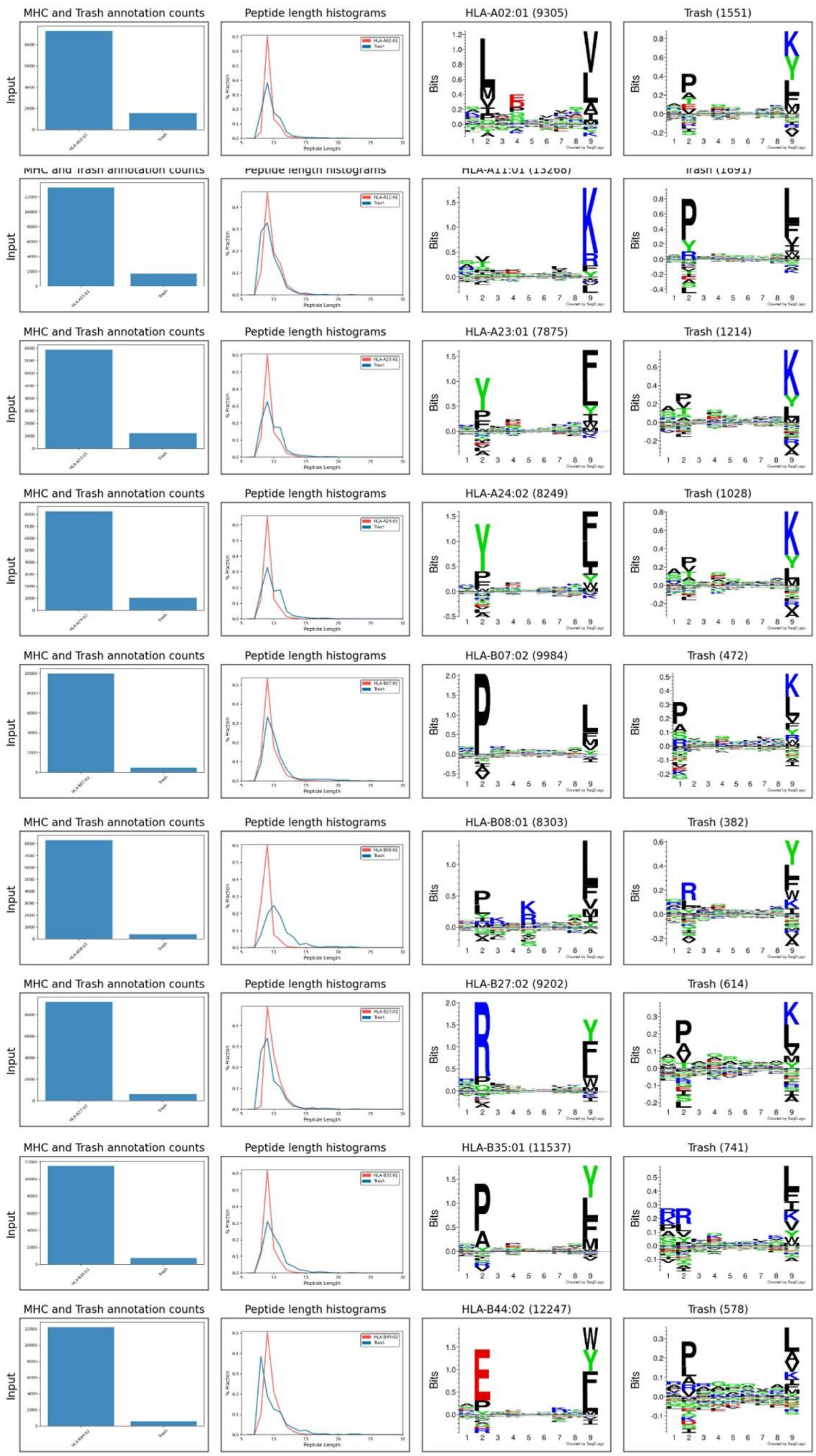

Supplementary Figure 9. CasΦ. Peptide length (6-30), excluding the use of the trash bin option. Peptides with a higher rank score than 20% are considered non-binder or contaminant and were assigned to the trash cluster.

SaCas9 – Extracted Overlap & Catalytic Residues

Sequence length: 1053 aa | Overlap residues: 23 | Catalytic residues: 3

Position	Amino Acid	One-Letter Code	Category
**10**	Aspartate	D	MAPPS and catalytic residue(s) overlap
**66**	Arginine	R	MAPPS and NA overlap
**69**	Lysine	K	MAPPS and NA overlap
**88**	Proline	P	MAPPS and NA overlap
**89**	Tyrosine	Y	MAPPS and NA overlap
**111**	Histidine	H	MAPPS and NA overlap
**114**	Lysine	K	MAPPS and NA overlap
**245**	Arginine	R	MAPPS and NA overlap
**248**	Lysine	K	MAPPS and NA overlap
**312**	Glycine	G	MAPPS and NA overlap
**313**	Tyrosine	Y	MAPPS and NA overlap
**314**	Arginine	R	MAPPS and NA overlap
**315**	Valine	V	MAPPS and NA overlap
**416**	Alanine	A	MAPPS and NA overlap
**419**	Asparagine	N	MAPPS and NA overlap
**459**	Lysine	K	MAPPS and NA overlap
**477**	Glutamate	E	MAPPS and catalytic residue(s) overlap
**557**	Histidine	H	MAPPS and catalytic residue(s) overlap
**651**	Tyrosine	Y	MAPPS and NA overlap
**654**	Arginine	R	MAPPS and NA overlap
**897**	Tyrosine	Y	MAPPS and NA overlap
**898**	Proline	P	MAPPS and NA overlap
**899**	Asparagine	N	MAPPS and NA overlap
**900**	Serine	S	MAPPS and NA overlap
**901**	Arginine	R	MAPPS and NA overlap
**904**	Valine	V	MAPPS and NA overlap

Colour Legend

(blue)	MAPPS and NA overlap residues
(peach)	MAPPS and catalytic residue(s) overlap

Supplementary Figure 10. SaCas9 - Extracted overlap and catalytic residues

AsCas12a – Extracted Overlap & Catalytic Residues

Sequence length: 1307 aa | Overlap residues: 45 | Catalytic residues: 1

Position	Amino Acid	One-Letter Code	Category
**14**	Serine	S	MAPPS and NA overlap
**15**	Lysine	K	MAPPS and NA overlap
**16**	Threonine	T	MAPPS and NA overlap
**18**	Arginine	R	MAPPS and NA overlap
**19**	Phenylalanine	F	MAPPS and NA overlap
**47**	Tyrosine	Y	MAPPS and NA overlap
**51**	Lysine	K	MAPPS and NA overlap
**134**	Lysine	K	MAPPS and NA overlap
**135**	Alanine	A	MAPPS and NA overlap
**166**	Threonine	T	MAPPS and NA overlap
**167**	Threonine	T	MAPPS and NA overlap
**174**	Glutamate	E	MAPPS and NA overlap
**175**	Asparagine	N	MAPPS and NA overlap
**176**	Arginine	R	MAPPS and NA overlap
**301**	Arginine	R	MAPPS and NA overlap
**306**	Phenylalanine	F	MAPPS and NA overlap
**307**	Lysine	K	MAPPS and NA overlap
**479**	Histidine	H	MAPPS and NA overlap
**511**	Leucine	L	MAPPS and NA overlap
**514**	Tyrosine	Y	MAPPS and NA overlap
**522**	Threonine	T	MAPPS and NA overlap
**523**	Lysine	K	MAPPS and NA overlap
**524**	Lysine	K	MAPPS and NA overlap
**530**	Lysine	K	MAPPS and NA overlap
**570**	Lysine	K	MAPPS and NA overlap
**574**	Arginine	R	MAPPS and NA overlap
**575**	Tyrosine	Y	MAPPS and NA overlap
**852**	Lysine	K	MAPPS and NA overlap
**862**	Arginine	R	MAPPS and NA overlap
**863**	Arginine	R	MAPPS and NA overlap
**864**	Phenylalanine	F	MAPPS and NA overlap
**870**	Phenylalanine	F	MAPPS and NA overlap
**938**	Phenylalanine	F	MAPPS and NA overlap
**940**	Tyrosine	Y	MAPPS and NA overlap
**962**	Glycine	G	MAPPS and NA overlap
**963**	Threonine	T	MAPPS and NA overlap
**964**	Isoleucine	I	MAPPS and NA overlap
**965**	Lysine	K	MAPPS and NA overlap
**966**	Aspartate	D	MAPPS and NA overlap
**967**	Leucine	L	MAPPS and NA overlap
**973**	Serine	S	MAPPS and NA overlap
**977**	Histidine	H	MAPPS and NA overlap
**993**	Glutamate	E	MAPPS and catalytic residue(s) overlap
**1013**	Glutamine	Q	MAPPS and NA overlap
**1014**	Glutamine	Q	MAPPS and NA overlap
**1017**	Lysine	K	MAPPS and NA overlap

Colour Legend

(blue)	MAPPS and NA overlap residues
(peach)	MAPPS and catalytic residue(s) overlap

Supplementary Figure 11. AsCas12a - Extracted overlap and catalytic residues

CasΦ – Extracted Overlap & Catalytic Residues

Sequence length: 757 aa | Overlap residues: 44 | Catalytic residues: 0

Position	Amino Acid	One-Letter Code	Category
8	Serine	S	MAPPS and NA overlap
10	Phenylalanine	F	MAPPS and NA overlap
11	Serine	S	MAPPS and NA overlap
14	Leucine	L	MAPPS and NA overlap
15	Lysine	K	MAPPS and NA overlap
138	Glycine	G	MAPPS and NA overlap
139	Arginine	R	MAPPS and NA overlap
142	Glycine	G	MAPPS and NA overlap
145	Lysine	K	MAPPS and NA overlap
146	Lysine	K	MAPPS and NA overlap
149	Leucine	L	MAPPS and NA overlap
270	Valine	V	MAPPS and NA overlap
273	Proline	P	MAPPS and NA overlap
274	Glycine	G	MAPPS and NA overlap
275	Leucine	L	MAPPS and NA overlap
276	Serine	S	MAPPS and NA overlap
277	Proline	P	MAPPS and NA overlap
278	Lysine	K	MAPPS and NA overlap
314	Arginine	R	MAPPS and NA overlap
315	Glycine	G	MAPPS and NA overlap
317	Leucine	L	MAPPS and NA overlap
318	Arginine	R	MAPPS and NA overlap
319	Asparagine	N	MAPPS and NA overlap
321	Arginine	R	MAPPS and NA overlap
322	Tryptophan	W	MAPPS and NA overlap
328	Lysine	K	MAPPS and NA overlap
364	Tyrosine	Y	MAPPS and NA overlap
367	Lysine	K	MAPPS and NA overlap
368	Tryptophan	W	MAPPS and NA overlap
371	Lysine	K	MAPPS and NA overlap
545	Lysine	K	MAPPS and NA overlap
547	Arginine	R	MAPPS and NA overlap
548	Leucine	L	MAPPS and NA overlap
553	Glutamine	Q	MAPPS and NA overlap
557	Asparagine	N	MAPPS and NA overlap
560	Leucine	L	MAPPS and NA overlap
561	Tryptophan	W	MAPPS and NA overlap
564	Lysine	K	MAPPS and NA overlap
565	Arginine	R	MAPPS and NA overlap
570	Tyrosine	Y	MAPPS and NA overlap
571	Leucine	L	MAPPS and NA overlap
572	Lysine	K	MAPPS and NA overlap
583	Arginine	R	MAPPS and NA overlap
586	Asparagine	N	MAPPS and NA overlap

Colour Legend

	MAPPS and NA overlap residues
	MAPPS and catalytic residue(s) overlap

Supplementary Figure 12. CasΦ - Extracted overlap and catalytic residues

Supplementary Tables

Supplementary Table 1. ELISA EC50 Values. Serial titrations of healthy donor serum ranging from 1:10, 1:20, 1:30, 1:40, 1:50, 1:75, and 1:100 were tested using ELISA for each Cas protein. The OD values were obtained; blank values were subtracted and the EC50 values were calculated using a nonlinear regression of serum dilution ELISA curves and are reported as the reciprocal serum dilution corresponding to half-maximal binding.

	SaCas9		AsCas12a		CasPhi	
Donor No.	EC50	R-Squared Value	EC50	R-Squared Value	EC50	R-Squared Value
1	8.68	0.996	15.80	0.987	6.57	0.991
2	4.45	0.943	19.60	0.949	11.12	0.997
3	16.60	0.914	16.98	0.981	5.18	0.997
4	7.90	0.983	9.58	0.995	18.72	0.982
5	13.05	0.996	29.86	0.980	13.64	0.990
6	22.14	0.983	8.62	0.969	27.42	0.994
7	30.24	0.978	35.43	0.947	48.82	0.983
8	16.02	0.930	10.90	0.972	87.95	0.968
9	13.50	0.992	4.65	0.959	17.25	0.957
10	9.73	0.991	14.23	0.981	7.49	0.991
11	12.71	0.966	15.13	0.938	49.25	0.989
12	5.82	0.993	2.06	0.964	7.03	0.992
13	19.59	0.979	18.23	0.981	3.69	0.988
14	15.81	0.991	11.75	0.982	6.36	0.975
15	8.99	0.986	5.33	0.959	4.74	0.949
16	75.71	0.934	9.61	0.942	15.34	0.921
17	2.59	0.955	9.54	0.981	5.46	0.951
18	14.39	0.979	5.56	0.995	2.52	0.989
19	8.45	0.973	5.65	0.980	325.60	0.966
20	12.39	0.943	0.02	0.937	4.42	0.996
21	5.43	0.965	8.74	0.980	4.03	0.967
22	53.10	0.964	28.78	0.955	22.91	0.983
23	3.69	0.982	2.43	0.984	0.07	0.959
24	40.33	0.978	18.53	0.980	26.54	0.991
25	11.34	0.994	5.35	0.969	10.57	0.998
26	3.64	0.985	16.88	0.977	9.00	0.995
27	17.54	0.995	26.00	0.919	12.58	0.997
28	8.82	0.986	10.40	0.976	10.02	0.996
29	8.32	0.997	6.52	0.989	23.74	0.985
30	8.17	0.991	6.43	0.994	18.76	0.997
31	15.11	0.996	42.03	0.971	43.28	0.993
32	11.04	0.997	9.83	0.936	3.30	0.991
33	19.15	0.995	8.87	0.923	6.28	0.995
34	12.31	0.943	36.51	0.955	7.61	0.983
35	0.01	0.993	25.70	0.959	2.98	0.983
36	3.41	0.995	2.20	0.963	14.69	0.978
37	7.95	0.991	0.00	0.995	4.08	0.997
38	8.08	0.994	5.41	0.988	10.40	0.993
39	28.54	0.905	7.03	0.998	10.32	0.989
40	10.94	0.997	3.42	0.998	8.96	0.986
41	12.08	0.967	8.48	0.995	8.90	0.993
42	14.38	0.969	40.98	0.995	5.08	0.996
43	20.84	0.984	9.34	0.982	4.87	0.994

Supplementary Table 2. Donor Demographics

Donor #	Age Range	Sex	Race	RACE CODES
1	25-30	Female	1	1 = European American
2	50-55	Male	2	2 = African American
3	25-30	Male	2	3 = Asian American
4	40-45	Female	2	4 = Central American
5	25-30	Female	1	5 = Native American
6	50-55	Female	1	6 = Unknown
7	50-55	Female	1	7 = Mixed Race
8	20-25	Female	1	8 = Other
9	55-60	Female	1	9 = Declined
10	25-30	Female	1	
11	50-55	Female	1	
12	20-25	Female	7	
13	50-55	Male	1	
14	40-45	Male	2	
15	45-50	Female	3	
16	35-40	Female	1	
17	20-25	Female	4	
18	65-70	Female	1	
19	65-70	Male	1	
20	45-50	Female	1	
21	40-45	Female	1	
22	20-25	Male	2	
23	45-50	Male	1	
24	30-35	Male	2	
25	60-65	Female	1	
26	30-35	Male	1	
27	25-30	Female	1	
28	30-35	Female	2	
29	60-65	Female	1	
30	60-65	Female	1	
31	55-60	Male	2	
32	50-55	Female	1	
33	50-55	Female	1	
34	30-35	Male	1	
35	50-55	Male	1	
36	35-40	Female	1	
37	50-55	Female	1	
38	50-55	Male	1	
39	60-65	Female	1	
40	60-65	Female	1	
41	35-40	Female	1	
42	20-25	Male	7	
43	60-65	Male	1	

Supplementary Table 3: Antibody dilutions and catalogue numbers

Antibody	Dilution	Source	Catalog No
HRP-conjugated goat anti-human IgG Fc secondary antibody	1 in 100000	Novus Bio	NB7449
HRP-conjugated anti-human IgG H&L	1 in 10000	Abcam	ab97175
APC-conjugated anti-human HLA-A, B, C antibody	1 in 20	Biolegend	311410
BD Horizon BV786 Mouse Anti-Human CD8	1 in 20	BD Biosciences	563823
α-SaCas9 (polyclonal antibody)	1 in 1000	Editas Medicine (gift)	N/A
α-AsCas12a (monoclonal antibody)	1 in 1000	Editas Medicine (gift)	N/A
α-CasΦ (monoclonal antibody)	1 in 1000	EnCor Biotechnology	MCA-5F95

Supplementary Table 4: Statistics

Figure	Comparisons	Test	*t/F*	*df*	95 % CI	*P* value
Figure 2.a	HSA vs SaCas9	Two-tailed Paired t-test	6.171	42	0.2131 to 0.4201	<0.0001
	HSA vs AsCas12a	Two-tailed Paired t-test	6.033	42	0.2081 to 0.4172	<0.0001
	HSA vs CasΦ	Two-tailed Paired t-test	8.116	42	0.2276 to 0.3783	<0.0001
	SaCas9 vs AsCas12a	Two-tailed Paired t-test	0.063	42	- 0.1294 to 0.1215	0.9499
	SaCas9 vs CasΦ	Two-tailed Paired t-test	0.213	42	- 0.1424 to 0.1152	0.8322
Figure 3.a	Unstimulated vs SaCas9	Two-tailed Paired t-test	5.961	42	33.20 to 67.17	<0.0001
	Unstimulated vs AsCas12a	Two-tailed Paired t-test	6.076	42	42.96 to 85.70	<0.0001
	Unstimulated vs CasΦ	Two-tailed Paired t-test	4.857	42	7.942 to 19.23	<0.0001
	SaCas9 vs CasΦ	Two-tailed Paired t-test	3.86	42	-55.73 to -17.46	0.0004
	SaCas9 vs AsCas12a	Two-tailed Paired t-test	1.153	42	-10.61 to 38.90	0.2554
	AsCas12a vs CasΦ	Two-tailed Paired t-test	4.734	42	29.11 to 72.37	<0.0001
