# Supplementary material for: Pre-existing antibody and T cell responses to SaCas9, AsCas12a and CasΦ are comparable in naïve individuals
Source: Nat Commun. 2026 Jul 1;17:8166. doi: 10.1038/s41467-026-75015-7 (PMC13457614; doi:10.1038/s41467-026-75015-7)
Supplement: Supplementary file 2 [file 41467_2026_75015_MOESM2_ESM.pdf]

nature portfolio

Corresponding author(s): Zuben E. Sauna
Elise Pepermans

Last updated by author(s): Jun 11, 2026

Reporting Summary

Nature Portfolio wishes to improve the reproducibility of the work that we publish. This form provides structure for consistency and transparency in reporting. For further information on Nature Portfolio policies, see our Editorial Policies and the Editorial Policy Checklist.

Statistics

For all statistical analyses, confirm that the following items are present in the figure legend, table legend, main text, or Methods section.

n/a	Confirmed	
☐	☒	The exact sample size (*n*) for each experimental group/condition, given as a discrete number and unit of measurement
☐	☒	A statement on whether measurements were taken from distinct samples or whether the same sample was measured repeatedly
☐	☒	The statistical test(s) used AND whether they are one- or two-sided<br>*Only common tests should be described solely by name; describe more complex techniques in the Methods section.*
☒	☐	A description of all covariates tested
☒	☐	A description of any assumptions or corrections, such as tests of normality and adjustment for multiple comparisons
☐	☒	A full description of the statistical parameters including central tendency (e.g. means) or other basic estimates (e.g. regression coefficient) AND variation (e.g. standard deviation) or associated estimates of uncertainty (e.g. confidence intervals)
☐	☒	For null hypothesis testing, the test statistic (e.g. F, t, r) with confidence intervals, effect sizes, degrees of freedom and P value noted<br>*Give P values as exact values whenever suitable.*
☒	☐	For Bayesian analysis, information on the choice of priors and Markov chain Monte Carlo settings
☒	☐	For hierarchical and complex designs, identification of the appropriate level for tests and full reporting of outcomes
☒	☐	Estimates of effect sizes (e.g. Cohen's d, Pearson's r), indicating how they were calculated

Our web collection on statistics for biologists contains articles on many of the points above.

Software and code

Policy information about availability of computer code

Data collection	Flow cytometry data collection was performed with BD FACSDiva (v6). Raw MS/MS data were analyzed using MSFragger within the FragPipe pipeline (https://fragpipe.nesvilab.org/) (version 22.0). CTL ImmunoSpot S6 Universal M2 Analyzer.
Data analysis	Flow Cytometry Data Analysis was performed with FlowJo (v10.5.3). SampPick Software to select a representative donor cohort can be found at https://www.github.com/fda/SampPick. Graph Pad Prism (v10).

For manuscripts utilizing custom algorithms or software that are central to the research but not yet described in published literature, software must be made available to editors and reviewers. We strongly encourage code deposition in a community repository (e.g. GitHub). See the Nature Portfolio guidelines for submitting code & software for further information.

Data

Policy information about availability of data

All manuscripts must include a data availability statement. This statement should provide the following information, where applicable:

- Accession codes, unique identifiers, or web links for publicly available datasets
- A description of any restrictions on data availability
- For clinical datasets or third party data, please ensure that the statement adheres to our policy

All data are included in the Supplementary Information or available from the authors, as are unique reagents used in this Article. The raw numbers for charts and graphs are available in the Source Data file whenever possible. The MAPPs Mass Spectrometry data generated in this study have been deposited in the PRIDE Database under accession code: PXD073207. The FACS data generated in this study have been deposited in the FLOW Repository under accession code: FR-FCM-

Z9WD.

Research involving human participants, their data, or biological material

Policy information about studies with human participants or human data. See also policy information about sex, gender (identity/presentation), and sexual orientation and race, ethnicity and racism.

Reporting on sex and gender	Sex and gender are not relevant to this study.
Reporting on race, ethnicity, or other socially relevant groupings	Not applicable
Population characteristics	Not applicable
Recruitment	The donor cohort used in the study was generated using the software SamPick to ensure a representation of HLA alleles that is comparable to the North American population.
Ethics oversight	NIH Blood Bank

Note that full information on the approval of the study protocol must also be provided in the manuscript.

Field-specific reporting

Please select the one below that is the best fit for your research. If you are not sure, read the appropriate sections before making your selection.

- [x] Life sciences
- [ ] Behavioural & social sciences
- [ ] Ecological, evolutionary & environmental sciences

For a reference copy of the document with all sections, see nature.com/documents/nr-reporting-summary-flat.pdf

Life sciences study design

All studies must disclose on these points even when the disclosure is negative.

Sample size	Donors for PBMCs (43) were collated from a larger pool of 150 MHC-typed donors using SampPick, an algorithm we developed recently (ref 32 in manuscript). SampPick optimizes cohort selection to closely match the frequency distribution of MHC variants in the target population; in this study that was the North American population.
Data exclusions	No data was excluded.
Replication	Replication was successful.
Randomization	Randomization was not relevant for this study.
Blinding	Blinding was not relevant for this study.

Reporting for specific materials, systems and methods

We require information from authors about some types of materials, experimental systems and methods used in many studies. Here, indicate whether each material, system or method listed is relevant to your study. If you are not sure if a list item applies to your research, read the appropriate section before selecting a response.

Materials & experimental systems

n/a	Involved in the study	
[ ]	[x]	Antibodies
[ ]	[x]	Eukaryotic cell lines
[x]	[ ]	Palaeontology and archaeology
[x]	[ ]	Animals and other organisms
[x]	[ ]	Clinical data
[x]	[ ]	Dual use research of concern
[x]	[ ]	Plants

Methods

n/a	Involved in the study	
[x]	[ ]	ChIP-seq
[ ]	[x]	Flow cytometry
[x]	[ ]	MRI-based neuroimaging

Antibodies

Antibodies used	HRP-conjugated goat anti-human IgG Fc secondary antibody (1 in 100000) - Novus Bio, Catalog number: NB7449<br>HRP-conjugated anti-human IgG H&L (1 in 10000)- Abcam, Catalog number: ab97175<br>APC-conjugated anti-human HLA-A, B, C antibody (1 in 20) - Biolegend, Catalog number: 311410

	BD Horizon BV786 Mouse Anti-Human CD8 (1 in 20) - BD Biosciences, Catalog number: 563823<br>α-SaCas9 (polyclonal antibody) (1 in 1000) - Editas Medicine, (gift)<br>α-AsCas12a (monoclonal antibody) (1 in 1000) - Editas Medicine (gift)<br>α-CasΦ (monoclonal antibody) (1 in 1000) - EnCor Biotechnology Catalog number: MCA-5F95
Validation	Antibodies were commercially available through BD, Jackson laboratory, MBL, Encore, Novus, Biolegend, Editas and previously published (according to manufacturers website).

Eukaryotic cell lines

Policy information about cell lines and Sex and Gender in Research

Cell line source(s)	Cell line sources were cited in the paper.
Authentication	Not applicable
Mycoplasma contamination	We did not check
Commonly misidentified lines (See ICLAC register)	Not applicable

Plants

Seed stocks	Not applicable
Novel plant genotypes	Not applicable
Authentication	Not applicable

Flow Cytometry

Plots

Confirm that:

- [x] The axis labels state the marker and fluorochrome used (e.g. CD4-FITC).
- [x] The axis scales are clearly visible. Include numbers along axes only for bottom left plot of group (a 'group' is an analysis of identical markers).
- [x] All plots are contour plots with outliers or pseudocolor plots.
- [x] A numerical value for number of cells or percentage (with statistics) is provided.

Methodology

Sample preparation	Donor PBMCs were isolated from NIH donor leucopaks. The cells were expanded for 20 days with peptides fro stimulation. After stimulation, the cells were collected, counted, and stained with amine reactive aqua live/dead stain for 30 mins followed by fc block, staining for surface markers and analyzed by flow cytometry.
Instrument	BD FACS Aria Fusion Sorter
Software	BD Flow Jo software
Cell population abundance	Abundance was determined by counting individual cells (events) using forward/side scatter or fluorescent markers and calculating their percentage or concentration relative to the total population.
Gating strategy	Antigen-specific populations were identified through sequential gating of lymphocytes, singlets, live cells, $CD8^+$ T-cell subset and tetramer-positive cells.

- [x] Tick this box to confirm that a figure exemplifying the gating strategy is provided in the Supplementary Information.
