# Supplementary material for: Pre-existing antibody and T cell responses to SaCas9, AsCas12a and CasΦ are comparable in naïve individuals
Source: Nat Commun. 2026 Jul 1;17:8166. doi: 10.1038/s41467-026-75015-7 (PMC13457614; doi:10.1038/s41467-026-75015-7)
Supplement: Supplementary file 3 [file 41467_2026_75015_MOESM3_ESM.pdf]

nature portfolio

Peer Review File

Pre-existing Antibody and T cell Responses to SaCas9, AsCas12a, and CasΦ Are Comparable in Naïve Individuals

Corresponding Author: Dr Zuben Sauna

This file contains all reviewer reports in order by version, followed by all author rebuttals in order by version.

Version 0:

Reviewer comments:

Reviewer #1

(Remarks to the Author)
Simhadri et al in "Immunogenicity Risk Assessment of SaCas9, AsCas12a and CasΦ" provide a concise but rigorous analysis of the potential presence of pre-existing immunity in a diverse human population to Cas proteins that might be predicted not to have pre-existing B and T-cell immunity because of their origin. The authors carefully show the surprising and significant result that there is both humoral and cellular immunity at equivalent frequencies to all three forms of Cas9. They then go on to map the epitopes that elicit T-cell immunity and demonstrate that for each protein, there are multiple epitopes that can elicit the T-cell response.

Overall, I find the work compelling and interesting and have the following suggestions that the authors should consider in improving it:

1. Would be very interesting to see if the overlap of immunity to 1, 2 or 3 of the Cas proteins in a single person. That is, if a person has immunity to one of the Cas is that independent or correlated to also having immunity to the others. Similarly, I assume but would be nice to see the data that if a person has antibodies against the Cas, what is the probability of the elispot being positive as well. And visa versa.
2. Textual change: The discussion should include the study from Missouri in dogs about the induction of immunity and clearance of cells transduced in the muscle using AAV delivery even though that used spCas9.
3. Textual Change: Suggest including in the discussion, that field awaits data from immune responses to Cas9 proteins given to humans. Especially those given multple doses. This includes both nucleases and base editors and especially "Baby KJ" who got three doses but as an infant.
4. Textual Change: The discussion would be helped by pointing out that since multiple epitopes can induce a T-cell response, it is unlikely that this response can be engineered around (unlike if it was a single epitope). This finding is consistent with what the Wagers and Church group found in mice treated with AAV deliverd spCas9. The potential induction of tolerance is fine.
5. Textual Change: The authors should include in the introduction that Cas proteins can be delivered by electroporation in the ex vivo setting with evidence that when RNP is used, the protein is usually not detectable at time of the cell infusion.

Reviewer #2

(Remarks to the Author)
This study examines pre-existing humoral and cellular responses to SaCas9, AsCas12a, and CasΦ in healthy donors and identifies MHC-I–presented peptides using mono-allelic lines. The topic is important, but several methodological and interpretative issues limit the strength of the conclusions. In its current form, the manuscript overextends population-level claims and “comparability/safer alternative” conclusions beyond what the data support. Specific comments are as follows;

1. The donor cohort (n=43) is too small to support population-level prevalence statements or broad generalizations about “North America.” Either (i) expand with an independent, larger cohort (≥100) and report confidence intervals, or (ii) remove population-level language and present the data descriptively.

2. Current ELISA design (single dilution, HSA-only negative, mean+3SD cut-point) is insufficient to claim seroprevalence. Buffer-match all antigens, perform serial titrations and report AUC/EC50, add irrelevant-protein and buffer-only controls, and

include an orthogonal confirmation (e.g., competition ELISA or Western blot). Provide endotoxin (EU/mg) and purity data for each protein preparation.

3. Claims that AsCas12a/CasΦ are "not safer" or have "comparable levels" are not supported without head-to-head statistical comparisons and functional data. Either provide functional evidence (e.g., serum inhibition of editing; CTL killing of editor-expressing cells) or limit the text to descriptive statements and remove the "not safer/comparable" language.

4. Protein sources and buffers differ between editors. Buffer-exchange into a common formulation before ELISA and restimulation, document adsorption/coating checks, and provide in-manuscript evidence of purity/identity (not only vendor statements).

Minor points:

-Clearly distinguish cultured versus ex vivo T-cell readouts in the Results section.

-Correct the stimulation index equation so it renders legibly and define variables.

-Provide donor demographics (age, sex, ethnicity) and explore associations with responses.

-Supply source data files (ELISA ODs, SFC counts, peptide lists with intensities/FDR).

-State explicitly that IgG was measured and discuss the absence of IgA/IgM analyses.

-Convert CRISPR/Cas9 to CRISPR-Cas9 throughtout the text

Reviewer #3

(Remarks to the Author)
This manuscript provides a comparative immunogenicity assessment of three CRISPR-associated nucleases—SaCas9, AsCas12a, and CasΦ, using pre-existing antibody profiling, T-cell ELISpot assays, and MHC-I–associated immunopeptidomics (MAPPs). The study addresses an important and timely question given the increasing clinical adoption of in vivo gene-editing therapeutics: specifically, whether non–pathogen-derived Cas orthologs (e.g., CasΦ from jumbo phage or AsCas12a from commensals) offer a reduced immunogenicity risk relative to SaCas9. The integration of donor pool stratification based on HLA allele distribution, paired analyses of humoral and cellular immunity, and development of a new intracellular expression–based MAPPs workflow represent strengths of the study.

However, although the topic is highly significant, the depth of mechanistic investigation falls short of what is expected for a high-impact journal. Several key conclusions are insufficiently supported, and opportunities for deeper analysis or validation are missing, particularly regarding functional T-cell immunogenicity and structural interpretation. Overall, the data are interesting and underscore the immunogenic potential of emerging Cas orthologs, but the current version remains descriptive rather than mechanistically rigorous.

Major Comments
1. Interpretation of T-cell responses and insufficient comparison across Cas proteins
While the authors report comparable levels of pre-existing antibodies across SaCas9, AsCas12a, and CasΦ, the T-cell responses differ notably. CasΦ elicits significantly lower IFN-γ SFCs than SaCas9 or AsCas12a, yet this important distinction is not addressed in the discussion or statistical interpretation. The current statement that "similar results" were obtained for all three proteins (Fig. 2C) is imprecise and potentially misleading. The comparison should not be only against unstimulated controls; direct cross-comparison among SaCas9, AsCas12a, and CasΦ is essential, especially given the biological argument that CasΦ might be less immunogenic. As shown, CasΦ responses were lower than SaCas9 ($P \leq 0.001$), but the statistical comparison with AsCas12a is missing or uncharacterized. These analyses should be provided. Additionally, the purity of recombinant SaCas9 and CasΦ proteins (>90%) raises concerns that contaminants may have contributed to background T-cell activation. Inclusion of a negative protein control produced by the same expression/purification process (e.g., human serum albumin) is necessary to rule out contamination-driven artefacts.

2. Relationship between MAPPs data and functional T-cell responses
The authors present MHC-I–associated peptides from all three Cas proteins, but the results are not integrated with the ELISpot findings. Given that both datasets aim to assess T-cell immunogenicity, correlations—or lack thereof—across donors or across protein epitopes should be addressed. Are peptides identified by MAPPs from each Cas protein enriched among donors with strong T-cell responses? Do CasΦ's fewer identified MHC-I peptides correspond to its reduced T-cell activation? Such analyses would substantially strengthen the mechanistic insight.
In addition, Fig. 3 is displayed at too low a resolution to allow proper assessment of peptide locations. A larger, high-resolution figure is needed.

3. Structural mapping and lack of follow-up functional validation

The superimposition of MHC-I peptides onto the 3D structures of SaCas9, AsCas12a, and CasΦ is conceptually interesting, particularly regarding proximity to DNA-binding or catalytic domains. However, the structural analysis remains superficial, and no functional experiments validate whether these peptides are immunogenic in T cells. At minimum, a subset of structurally highlighted peptides should be evaluated in vitro for their ability to activate CD8+ T cells, and whether they represent dominant or subdominant epitopes. Furthermore, for a journal at the level of Nature Communications, the study would be significantly strengthened by exploring deimmunization strategies, such as identifying naturally occurring or engineered CasΦ variants with reduced epitope presentation but retained function. Without this, the structural analysis feels incomplete and lacks translational relevance.

Version 1:

Reviewer comments:

Reviewer #1

(Remarks to the Author)
The authors have adequately addressed all of my concerns. It seems that the authors have also addressed the other reviewers concerns but I will defer to them as to whether the authors have done so adequately. The surprising finding that humans have both humoral and cellular immunity to Cas proteins that are not known to derive from organisms that infect or colonize humans is significant enough in my judgement to warrant publication in Nature Communications. The genome editing field still seems to underestimate the importance of this finding which also makes this work significant.

Reviewer #2

(Remarks to the Author)
I thank the authors for the careful revision. The manuscript is substantially improved, and my major concerns have been satisfactorily addressed. The added serial ELISA titrations, EC50 values, immunoblot confirmation, protein purity/endotoxin data, donor-level B- and T-cell summaries.

I have only minor remaining comments:

Please slightly soften the Abstract/Discussion language stating that AsCas12a and CasΦ are not less immunogenic alternatives to Cas9. The data support substantial immune recognition in this cohort, but not definitive clinical equivalence in immunogenicity risk.

Reviewer #3

(Remarks to the Author)
The authors have provided responses to my questions; however, in my view, several key points remain only partially addressed, and further improvement is still required.

Regarding my first question on the comparison between SaCas9, AsCas12a and CasΦ in Figure 2, the authors have now added statistical analyses showing that CasΦ induced significantly lower positive IFN-γ ELISpot responses compared with SaCas9 and AsCas12a. Although this difference is mentioned in the Discussion (Line 281), it is not accurately reflected in the Abstract, which states: “We demonstrate that SaCas9, AsCas12a and CasΦ have comparable levels of pre-existing and T-cell responses.” Given the new statistical analysis, this statement appears inaccurate and should be revised to better reflect the data.

Regarding my second question, I appreciate the authors’ efforts to test one specific peptide in the context of the corresponding HLA type. However, I am not fully convinced by the authors’ statement that “a comprehensive study along these lines is outside the scope of this revision,” particularly as they also acknowledge that assessing the concordance between MAPPs data and functional T-cell responses would be informative. Even if a comprehensive analysis is not feasible, additional validation or a clearer discussion of this limitation would strengthen the study.

Regarding my third question, the additional information on structural prediction is helpful. However, such predictions can be uncertain and do not, by themselves, fully address the concern that prompted my suggestion for de-immunisation validation. Since this experimental validation has not been performed, the conclusion remains somewhat under-supported.

Overall, the topic is interesting and potentially important. However, I feel that the accuracy of some statements and the depth of experimental validation need to be substantially improved.

Open Access This Peer Review File is licensed under a Creative Commons Attribution 4.0 International License, which permits use, sharing, adaptation, distribution and reproduction in any medium or format, as long as you give appropriate credit to the original author(s) and the source, provide a link to the Creative Commons license, and indicate if changes were made.
In cases where reviewers are anonymous, credit should be given to 'Anonymous Referee' and the source.
The images or other third party material in this Peer Review File are included in the article's Creative Commons license, unless indicated otherwise in a credit line to the material. If material is not included in the article's Creative Commons license and your intended use is not permitted by statutory regulation or exceeds the permitted use, you will need to obtain permission directly from the copyright holder.
To view a copy of this license, visit https://creativecommons.org/licenses/by/4.0/

Responses to reviewers' comments

NOTE: The figure and line numbers indicated in our responses to the reviewers align with the clean version of the revised manuscript.

<u>Reviewer #1:</u>

Simhadri et al in "Immunogenicity Risk Assessment of SaCas9, AsCas12a and Cas?" provide a concise but rigorous analysis of the potential presence of pre-existing immunity in a diverse human population to Cas proteins that might be predicted not to have pre-existing B and T-cell immunity because of their origin. The authors carefully show the surprising and significant result that there is both humoral and cellular immunity at equivalent frequencies to all three forms of Cas9. They then go on to map the epitopes that elicit T-cell immunity and demonstrate that for each protein, there are multiple epitopes that can elicit the T-cell response.

Overall, I find the work compelling and interesting and have the following suggestions that the authors should consider in improving it:

Q1.1. Would be very interesting to see if the overlap of immunity to 1, 2 or 3 of the Cas proteins in a single person. That is, if a person has immunity to one of the Cas is that independent or correlated to also having immunity to the others. Similarly, I assume but would be nice to see the data that if a person has antibodies against the Cas, what is the probability of the elispot being positive as well. And visa versa.

Response: As the same donor set was used to evaluate all 3 Cas proteins it is possible to address the reviewer's question. We have now included figures that permit visual comparison of identification pre-existing B- and T-cell immunity to each of the 3 Cas proteins (**Figures 2b, 2f**) for each donor. We have also provided a summary figure showing the percentage of donors who exhibit both a T- and B-cell response to all 3 Cas proteins (**Figure 2g**). With respect to a humoral immunity, a MHC-engagement and T-cell proliferation are both necessary but not sufficient conditions for the development of antibodies. Consistent with this paradigm, for all three Cas proteins; more donors show a T-cell response (**Figure 2e**) than preexisting antibodies (**Figure 2b**).

Q1.2. Textual change: The discussion should include the study from Missouri in dogs about the induction of immunity and clearance of cells transduced in the muscle using AAV delivery even though that used spCas9.

Response: Thank you for bringing this important study to our attention. The report has been discussed in the context of the current manuscript (lines 312-321).

Q1.3. Textual Change: Suggest including in the discussion, that field awaits data from immune responses to Cas9 proteins given to humans. Especially those given mulitple doses. This includes both nucleases and base editors and especially "Baby KJ" who got three doses but as an infant.

Response: We agree that this is an important caveat, and we have discussed it in the revised manuscript (lines 322-325).

Q1.4. Textual Change: The discussion would be helped by pointing out that since multiple epitopes can induce a T-cell response, it is unlikely that this response can be engineered around (unlike if it was a single epitope). This finding is consistent with what the Wagers and Church group found in mice treated with AAV deliverd spCas9. The potential induction of tolerance is fine.

Response: The revised discussion includes this statement (lines 345-358). In addition, the discussion of the placement of the epitopes in the structures of the Cas proteins also includes subtle differences between the three Cas proteins vis-à-vis engineering for de-immunization (lines 358-364).

Q1.5. Textual Change: The authors should include in the introduction that Cas proteins can be delivered by electroporation in the ex vivo setting with evidence that when RNP is used, the protein is usually not detectable at time of the cell infusion.

Response: The discussion includes this statement (lines 355-358).

Reviewer #2 (Gene therapy, CRISPR/Cas9) (Remarks to the Author):

This study examines pre-existing humoral and cellular responses to SaCas9, AsCas12a, and Cas? in healthy donors and identifies MHC-I presented peptides using mono-allelic lines. The topic is important, but several methodological and interpretative issues limit the strength of the conclusions. In its current form, the manuscript overextends population-level claims and comparability/safer alternative conclusions beyond what the data support. Specific comments are as follows:

Q2.1. The donor cohort (n=43) is too small to support population-level prevalence statements or broad generalizations about North America. Either (i) expand with an independent, larger cohort (?100) and report confidence intervals, or (ii) remove population-level language and present the data descriptively.

Response: Per the reviewer's suggestion we have removed population level statements. We moreover agree with you that given the diversity of HLA variants in populations, even 100 donors would not accurately reflect the population. This is why we previously developed an algorithm (*McGill, Yogurtcu, Yang, Verthelyi, & Sauna. Front. Immunol. 10: 2894*; reference **32** in the manuscript) that utilizes simulated annealing to optimizes a cohort to match the HLA distribution of the target population. When used to select a cohort of 50 donors this algorithm performed 7 standard deviations better than 10 million randomly picked cohorts. In the manuscript we have explained how our donor cohort was improved using this algorithm (lines 365-367).

Q2.2. Current ELISA design (single dilution, HSA-only negative, mean+3SD cut-point) is insufficient to claim seroprevalence. Buffer-match all antigens, perform serial titrations and report AUC/EC50, add irrelevant-protein and buffer-only controls, and include an orthogonal confirmation (e.g., competition ELISA or Western blot). Provide endotoxin (EU/mg) and purity data for each protein preparation.

Response: As pre the reviewer's suggestion we have performed the ELISA using serial dilutions of donor sera and reported EC50 values for each donor (see **Supplementary Table 1, Figure 2** and lines 88-89 in the text). We agree with the reviewer that the results would benefit from an orthogonal method and have carried out immuno-blots. **Figure 2b** shows the response of each donor with vis-à-vis the ELISA and immuno-blot. We have also reported the percentage of donors that are seropositive for each of the Cas proteins based on the conservative criterion of testing positive in both assays (ELISA and immuno-blot). Endotoxin levels and purity data for all three Cas proteins are provided in **Supplementary Figure 1**.

Q2.3. Claims that AsCas12a/Cas? are not safer or have comparable levels are not supported without head-to-head statistical comparisons and functional data. Either provide functional evidence (e.g., serum inhibition of editing; CTL killing of editor-expressing cells) or limit the text to descriptive statements and remove the not safer/comparable language.

Response: We agree with the reviewer's critique and have removed statements about the relative safety of the Cas proteins.

Q2.4. Protein sources and buffers differ between editors. Buffer-exchange into a common formulation before ELISA and restimulation, document adsorption/coating checks, and provide in-manuscript evidence of purity/identity (not only vendor statements).

Response: We believe that buffer exchange was not necessary because all proteins are diluted at least 2,000-fold into a common buffer for the experiments. Purity data for all three Cas proteins can be seen in **Supplementary Figure 1**.

Q2.5. Minor points:

-Clearly distinguish cultured versus ex vivo T-cell readouts in the Results section.

Response: The revised Results distinguishes between cultured and ex vivo T cell readouts (lines 124-184).

-Correct the stimulation index equation so it renders legibly and define variables.

Response: The stimulation index is now visible clearly and all variables have been defined (lines 473).

-Provide donor demographics (age, sex, ethnicity) and explore associations with responses.

Response: Donor demographics have been reported (Supplementary Table 2). There were no clear associations between these characteristics and responses.

-Supply source data files (ELISA ODs, SFC counts, peptide lists with intensities/FDR).

Response: Source data files have been uploaded to Harvard Dataverse. https://dataverse.harvard.edu/previewurl.xhtml?token=fe04cd6a-4e79-42fa-9190-db447e40690e

-State explicitly that IgG was measured and discuss the absence of IgA/IgM analyses.

Response: We have stated that IgG was measured, and the absence of IgA and IgM analyses listed as a limitation of the study (line 369).

-Convert CRISPR/Cas9 to CRISPR-Cas9 throughout the text

Response: We have used the term CRISPR-Cas9 consistently.

Reviewer #3 (B cell immunity) (Remarks to the Author):

This manuscript provides a comparative immunogenicity assessment of three CRISPR-associated nucleases SaCas9, AsCas12a, and Cas?, using pre-existing antibody profiling, T-cell ELISpot assays, and MHC-I associated immunopeptidomics (MAPPs). The study addresses an important and timely question given the increasing clinical adoption of in vivo gene-editing therapeutics: specifically, whether non pathogen-derived Cas orthologs (e.g., Cas? from jumbo phage or AsCas12a from commensals) offer a reduced immunogenicity risk relative to SaCas9. The integration of donor pool stratification based on HLA allele distribution, paired analyses of humoral and cellular immunity, and development of a new intracellular expression based MAPPs workflow represent strengths of the study. However, although the topic is highly significant, the depth of mechanistic investigation falls short of what is expected for a high-impact journal. Several key conclusions are insufficiently supported, and opportunities for deeper analysis or validation are missing, particularly regarding functional T-cell immunogenicity and structural interpretation. Overall, the data are interesting and underscore the immunogenic potential of emerging Cas orthologs, but the current version remains descriptive rather than mechanistically rigorous.

Major Comments

Q3.1. Interpretation of T-cell responses and insufficient comparison across Cas proteins While the authors report comparable levels of pre-existing antibodies across SaCas9, AsCas12a, and Cas?, the T-cell responses differ notably. Cas? elicits significantly lower IFN-? SFCs than SaCas9 or AsCas12a, yet this important distinction is not addressed in the discussion or statistical interpretation. The current statement that similar results were obtained for all three proteins (Fig. 2C) is imprecise and potentially misleading. The comparison should not be only against unstimulated controls; direct cross-comparison among SaCas9, AsCas12a, and Cas? is essential, especially given the biological argument that Cas? might be less immunogenic. As shown, Cas? responses were lower than SaCas9 (P ? 0.001), but the statistical comparison with AsCas12a is missing or uncharacterized. These analyses should be provided.

Response: Consistent with Reviewer 2's critique the revised manuscript does not have any broad statements about the relative clinical safety of the Cas proteins. However, we take Reviewer 3's point about a more thorough analyses and discussion of the differences with respect to the in vitro responses to the 3 Cas proteins. We have provided cross-comparisons between SaCas9, AsCas12a, and CasΦ in **Figure 2** and discussed these in the text (lines 276-282).

Additionally, the purity of recombinant SaCas9 and Cas? proteins (>90%) raises concerns that contaminants may have contributed to background T-cell activation. Inclusion of a negative protein control produced by the same expression/purification process (e.g., human serum albumin) is necessary to rule out contamination-driven artefacts.

Response: Our protein preparations are of very high quality and free of endotoxin. Please see Endotoxin levels and purity data for all three Cas proteins which are provided in **Supplementary Figure 1**.

Q3.2. Relationship between MAPPs data and functional T-cell responses. The authors present MHC-I associated peptides from all three Cas proteins, but the results are not integrated with the ELISpot findings. Given that both datasets aim to assess T-cell immunogenicity, correlations or lack thereof across donors or across protein epitopes should be addressed. Are peptides identified by MAPPs from each Cas protein enriched among donors with strong T-cell responses? Do Cas? s fewer identified MHC-I peptides correspond to its reduced T-cell activation? Such analyses would substantially strengthen the mechanistic insight. In addition, Fig. 3 is displayed at too low a resolution to allow proper assessment of peptide locations. A larger, high-resolution figure is needed.

Response: We agree with the reviewer that a study showing the level of concordance between MAPPs data and functional responses is warranted. A comprehensive study along these lines is outside the scope of this revision. However, we have carried out a proof-of-principle study: Peptide GVYKFVTVK was identified on a monoallelic cell line expressing HLA*A11:01 in the MAPPs assay. We used this peptide to stimulate and expand Donor PBMCs of the same HLA variant (A11:01) for 20 days. We also generated a tetramer using A11:01 and the GVYKFVTVK. This tetramer was used to identify CD8+ T-Cells in the expanded PBMC population. We demonstrate a significant increase in the % of $CD8^+$ $Tetramer^+$ T-cells after a 20-day expansion with peptide GVYKFVTVK in 4 independent donors. The data are presented in **Figure 4** in the revised manuscript and described in the results section (lines 190-211).

Q3.3. Structural mapping and lack of follow-up functional validation. The superimposition of MHC-I peptides onto the 3D structures of SaCas9, AsCas12a, and Cas? is conceptually interesting, particularly regarding proximity to DNA-binding or catalytic domains. However, the structural analysis remains superficial, and no functional experiments validate whether these peptides are immunogenic in T cells. At minimum, a subset of structurally highlighted peptides should be evaluated in vitro for their ability to activate CD8+ T cells, and whether they represent dominant or subdominant epitopes. Furthermore, for a journal at the level of Nature Communications, the study *would be significantly strengthened* by exploring

deimmunization strategies, such as identifying naturally occurring or engineered Cas? variants with reduced epitope presentation but retained function. Without this, the structural analysis feels incomplete and lacks translational relevance.

Response: In the revised manuscript we have significantly expanded the structural mapping and analyses (see revised **Figure 5** and lines 224-247). We have mapped the peptides identified in the MAPPs assay on the structures of the three Cas proteins and identified the peptides that are likely to be at positions that are functionally important (**Figure 5**). We agree with the reviewer that a de-immunization study is important. However, that is an independent study that we are currently completing. Nonetheless we have discussed the implications of structural mapping on de-immunization of the three Cas proteins (lines 360-364).

POINT-BY-POINT RESPONSES TO REVIEWERS' COMMENTS

Reviewer #1 (Remarks to the Author):

The authors have adequately addressed all of my concerns. It seems that the authors have also addressed the other reviewers concerns but I will defer to them as to whether the authors have done so adequately. The surprising finding that humans have both humoral and cellular immunity to Cas proteins that are not known to derive from organisms that infect or colonize humans is significant enough in my judgement to warrant publication in Nature Communications. The genome editing field still seems to underestimate the importance of this finding which also makes this work significant.

Response: We thank the reviewer for their comments. There are no unresolved questions.

Reviewer #2 (Remarks to the Author):

I thank the authors for the careful revision. The manuscript is substantially improved, and my major concerns have been satisfactorily addressed. The added serial ELISA titrations, EC50 values, immunoblot confirmation, protein purity/endotoxin data, donor-level B- and T-cell summaries.

I have only minor remaining comments:

Please slightly soften the Abstract/Discussion language stating that AsCas12a and CasΦ are not less immunogenic alternatives to Cas9. The data support substantial immune recognition in this cohort, but not definitive clinical equivalence in immunogenicity risk.

Response: We agree with the reviewer and have changed the language in the revised manuscript (see Lines 18-20, 324 -355).

Reviewer #3 (Remarks to the Author):

The authors have provided responses to my questions; however, in my view, several key points remain only partially addressed, and further improvement is still required.

Regarding my first question on the comparison between SaCas9, AsCas12a and CasΦ in Figure 2, the authors have now added statistical analyses showing that CasΦ induced significantly lower positive IFN-γ ELISpot responses compared with SaCas9 and AsCas12a. Although this difference is mentioned in the Discussion (Line 281), it is not accurately reflected in the Abstract, which states: “We demonstrate that SaCas9, AsCas12a and CasΦ have comparable levels of pre-existing and T-cell responses.” Given the new statistical analysis, this statement appears inaccurate and should be revised to better reflect the data.

Response: We thank the reviewer for identifying this inconsistency and have rectified it (Lines 10 -20).

Regarding my second question, I appreciate the authors’ efforts to test one specific peptide in the context of the corresponding HLA type. However, I am not fully convinced by the authors’ statement that “a comprehensive study along these lines is outside the scope of this revision,” particularly as they also acknowledge that assessing the concordance between MAPPs data and functional T-cell responses would be informative. Even if a comprehensive analysis is not feasible, additional validation or a clearer discussion of this limitation would strengthen the study.

Response: We take the reviewer's point and have explicitly discuss the limitation of the study (Lines 324 -355). We would also like to assure the reviewer that an additional study to fully assess the concordance between MAPPs data and T-cell responses is underway.

Regarding my third question, the additional information on structural prediction is helpful. However, such predictions can be uncertain and do not, by themselves, fully address the concern that prompted my suggestion for de-immunisation validation. Since this experimental validation has not been performed, the conclusion remains somewhat under-supported.

Response: We recognize the need for a de-immunization study and are in the process of submitting such a study. As a de-immunization study requires: (i) Careful selection of the de-immunization locations. (ii) Identification of donor population with appropriate HLA variants. (iii) Demonstration of successful de-immunization by orthologous techniques. (iv) Careful characterization of de-immunized variants with respect to functionality. (v) Comparison of off-target editing by wild-type and de-immunized Cas variants. Consequently, we are submitting the de-immunization study as an independent manuscript rather than an add-on to the current study.

Overall, the topic is interesting and potentially important. However, I feel that the accuracy of some statements and the depth of experimental validation need to be substantially improved.
